# UV damage induces G3BP1-dependent stress granule formation that is not driven by mTOR inhibition-mediated translation arrest

**DOI:** 10.1242/jcs.248310

**Published:** 2020-10-28

**Authors:** Shan Ying, Denys A. Khaperskyy

**Affiliations:** Department of Microbiology and Immunology, Dalhousie University, Halifax, NS B3H 4R2, Canada

**Keywords:** Stress granule, UVC, GCN2, G3BP1, mTOR

## Abstract

Translation arrest is a part of the cellular stress response that decreases energy consumption and enables rapid reprioritisation of gene expression. Often translation arrest leads to condensation of untranslated messenger ribonucleoproteins (mRNPs) into stress granules (SGs). Studies into mechanisms of SG formation and functions are complicated because various types of stress cause formation of SGs with different properties and composition. In this work, we focused on the mechanism of SG formation triggered by UV damage. We demonstrate that UV-induced inhibition of translation does not involve inhibition of the mechanistic target of rapamycin (mTOR) signaling or dissociation of the 48S preinitiation complexes. The general control non-derepressible 2 (GCN2; also known as EIF2AK4) kinase contributes to UV-induced SG formation, which is independent of the phosphorylation of the eukaryotic translation initiation factor 2α. Like many other types of SGs, condensation of UV-induced granules requires the Ras-GTPase-activating protein SH3-domain-binding protein 1 (G3BP1). Our work reveals that, in UV-treated cells, the mechanisms of translation arrest and SG formation may be unlinked, resulting in SGs that do not contain the major type of polysome-free preinitiation complexes that accumulate in the cytoplasm.

This article has an associated First Person interview with the first author of the paper.

## Abstract (precis)

**Summary:** The catalytic activity of GCN2 is important for G3BP1-dependent stress granule formation in response to UV damage, but neither eIF2α phosphorylation nor mTOR inhibition are involved in the mechanism.

## INTRODUCTION

Inhibition of translation initiation in response to various types of stress leads to ribosome runoff from mRNAs and condensation of untranslated mRNA–protein complexes (messenger ribonucleoproteins; mRNPs) into large foci called stress granules (SGs) (Protter and Parker, 2016). SGs are cytoplasmic phase-separated organelles that accumulate polysome-free mRNPs and dozens of proteins and other molecules that are held together by multiple weak RNA–protein and protein–protein interactions (Kedersha et al., 2013; Lin et al., 2015; Protter and Parker, 2016). Assembly of SGs is driven by the SG-nucleating proteins, including the Ras-GTPase-activating protein SH3-domain-binding proteins 1 and 2 (G3BP1 and G3BP2; hereafter G3BP1/2), T-cell internal antigen 1 (TIA-1) and T-cell internal antigen related (TIAR, also known as TIAL1), which are often used as nearly universal SG markers. SGs are believed to play a role in regulating stress responses; however, the molecular functions of SG formation remain poorly understood (Kedersha et al., 2013; McCormick and Khaperskyy, 2017).

In some cases, aberrant SG dynamics can contribute to neurodegeneration, because phase separation facilitates formation of stable cytotoxic aggregates of mutant proteins linked to neurodegenerative diseases (reviewed in Baradaran-Heravi et al., 2020; Li et al., 2013; Monahan et al., 2016). However, transient translation arrest and SG formation are most often discussed in the context of the pro-survival integrated stress response (ISR) programme triggered by phosphorylation of eukaryotic initiation factor 2α (eIF2α) on serine-51 by one of the four kinases activated by different types of stress (Anderson and Kedersha, 2002; Kedersha et al., 2002, 2013). The heme-regulated inhibitor (HRI, also known as EIF2AK1) kinase is activated by oxidative stress (McEwen et al., 2005); the general control non-derepressible 2 (GCN2, also known as EIF2AK4) is activated by amino acid starvation (Wek et al., 1995) or UV damage (Deng et al., 2002); the double-stranded RNA (dsRNA)-activated protein kinase (PKR; also known as EIF2AK2) is activated by viral dsRNA replication intermediates (García et al., 2007); and the PKR-like endoplasmic reticulum kinase (PERK; also known as EIF2AK3) is activated by endoplasmic reticulum stress (Harding et al., 2000a). When eIF2α is phosphorylated, it stably binds eIF2B and prevents it from mediating GDP to GTP exchange, which is required for generation of the translation initiation competent eIF2–GTP–Met-tRNA^Met^ ternary complex (Jackson et al., 2010). This inhibits translation initiation downstream of the assembly of the 48S translation preinitiation complex, which includes the eIF4F complex (consisting of the eIF4E cap binding protein, eIF4G scaffolding subunit, and eIF4A RNA helicase; note there is more than one isoform of eIF4G and eIF4A in mammals) bound to the mRNA 5′ m^7^GTP cap and the 43S small ribosomal subunit (Jackson et al., 2010). Consequently, components of the 48S pre-initiation complexes become the major constituents of SGs that form (Kedersha et al., 2002).

The phospho-eIF2α-dependent translation arrest and SG formation can be blocked by pharmacological inhibition or interference with the expression of specific eIF2α kinases (Aulas et al., 2017), as well as by genetic replacement of the wild-type (WT) eIF2α gene with an unphosphorylatable S51A mutant (Scheuner et al., 2001). Recently, a small-molecule ISR inhibitor (ISRIB) was developed (Sidrauski et al., 2013), which does not interfere with eIF2α phosphorylation, but instead blocks its effect on translation initiation by facilitating eIF2B-mediated GDP to GTP exchange (Sidrauski et al., 2015). When SG formation is dependent on eIF2α phosphorylation, for example, in response to treatments with sodium arsenite (As; induces oxidative stress and HRI activation) or thapsigargin (Tg; induces endoplasmic reticulum stress and PERK activation), it is strongly inhibited by ISRIB (Sidrauski et al., 2015).

SG formation can also be phospho-eIF2α independent. For example, oxidative stress caused by hydrogen peroxide (H_2_O_2_) or sodium selenite (Se) induces SGs through a 4E-binding protein (EIF4EBP1–EIF4EBP3 proteins generically referred to as 4E-BP)-dependent mechanism via inhibition of mechanistic target of rapamycin (mTOR) (Emara et al., 2012; Fujimura et al., 2012). Under conditions that favour growth and proliferation of cells, mTOR phosphorylates 4E-BP on multiple serine/threonine residues (Brunn et al., 1997; Sun et al., 2019). Hyperphosphorylated 4E-BP cannot bind eIF4E, allowing for eIF4E–eIF4G complex formation and assembly of eIF4F. Inhibition of mTOR leads to rapid 4E-BP dephosphorylation and binding and sequestration of eIF4E away from eIF4G. Under these conditions, 48S preinitiation complexes cannot form, and translation initiation is inhibited (Thoreen et al., 2012). SGs that form via a 4E-BP-dependent mechanism lack subunits of eIF3 (e.g. eIF3B) because eIF4F complex formation is required for recruitment of eIF3 to the mRNA (Emara et al., 2012).

One of the stress stimuli that induces SG formation is exposure to UV light. UV exposure is ubiquitous for all living organisms exposed to sunlight. Although high energy UVC (<290 nm wavelength) radiation from the sun is blocked by the ozone layer in the atmosphere, the most dangerous spectrum of UV light that reaches the surface and contributes to the development of skin cancer, UVB (290–320 nm), causes the same types of DNA damage as UVC (Cadet and Douki, 2018). Accordingly, many studies analysing the effects of UV light on living cells, including our present study, utilize standard 254 nm UVC bulbs. Historically, UV radiation-induced DNA damage and cell cycle arrest has been studied extensively because of their relationship to carcinogenesis (Jhappan et al., 2003). By comparison, UV-induced translation arrest and SG formation, and their roles in cell survival following UV damage, remain poorly understood. Several studies examining UV-induced SGs have revealed that despite causing robust activation of GCN2 and GCN2-mediated eIF2α phosphorylation, UV-induced SG formation does not depend on phospho-eIF2α (Aulas et al., 2017; Moutaoufik et al., 2014). The UV-induced granules are true SGs because their formation can be inhibited by cycloheximide (CHX) and they accumulate G3BP1/2, TIA-1, TIAR and FMRP. However, like SGs caused by H_2_O_2_, UV-induced SGs do not accumulate eIF4G and eIF3B, and poorly recruit PABP1 and poly(A) RNA (Aulas et al., 2017; Moutaoufik et al., 2014). Whether the mechanism of SG formation in response to UV damage is also similar to that of H_2_O_2_ or Se-induced SGs and involves mTOR inhibition has not been investigated.

In this work, we systematically examined translation arrest and SG formation in human U2OS osteosarcoma cells in response to UV light. We demonstrate that GCN2-mediated eIF2α phosphorylation is responsible for some but not all the UV-induced translation inhibition. By contrast, we did not reveal any contribution of mTOR inhibition or disassembly of eIF4F complex to the translation arrest following UV exposure. By performing a Me^7^GTP–agarose pulldown assay and co-immunoprecipitation with eIF3B-specific antibody, we demonstrate that the bulk of 48S preinitiation complexes remains intact even when translation is strongly inhibited by UV light. Our studies reveal that UV damage triggers a novel mechanism of SG formation that relies neither on eIF2α phosphorylation nor mTOR inhibition. UV-induced SG condensation is driven by G3BP1, it is enhanced by the catalytic activity of GCN2, but recruits only a small fraction of untranslated mRNPs that lose their association with eIF4G and eIF3B.

## RESULTS

### UV-induced SG formation is not associated with inhibition of mTOR signalling

**Fig. 1.**
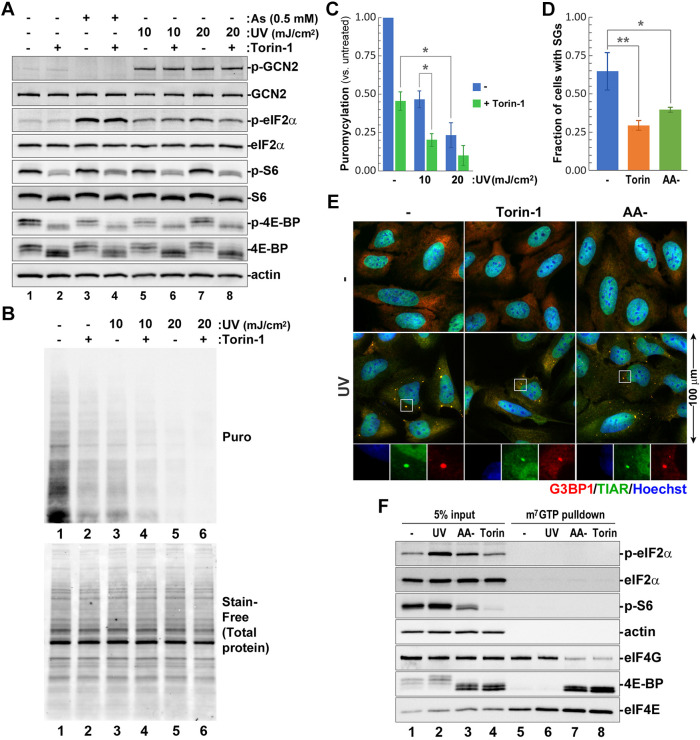
**Translation arrest and SG formation in response to UV damage is independent of mTOR inhibition.** (A,B) U2OS cells were exposed to the indicated doses of UV light and incubated for 2 h with or without Torin-1 prior to analysis. (A) Phosphorylation of GCN2, eIF2α, and the mTOR substrates 4E-BP and S6 were analysed by western blotting. Lysates from cells treated with 0.5 mM sodium arsenite (As) were included for comparison. Staining for actin was used as loading control. (B) Protein synthesis rates were analysed through the ribopuromycylation assay and by western blotting with anti-puromycin antibody (Puro, top panel). Total protein content in each lane was visualised using Stain-Free reagent (bottom panel). (C) Translation arrest was quantified (mean±s.d.) from ribopuromycylation assays represented in B (*n*=3). *P<0.05 (one-way ANOVA followed by Tukey's multiple comparisons test). (D,E) UV-induced SG formation (20 mJ/cm^2^) was analysed in control cells or cells incubated in amino acid-free medium (AA-) or Torin-1-containing medium. (D) SG formation was quantified (mean±s.d.) in cells based on immunofluorescence staining (*n*=3). *P<0.05, **P<0.01 (two-way ANOVA followed by Tukey's multiple comparisons test). (E) Representative immunofluorescence staining for G3BP1 (red) and TIAR (green). Nuclei were stained with Hoechst dye (blue). (F) eIF4E–eIF4G and eIF4E–4E-BP interactions in cells treated with UV light (UV), amino acid-free medium (AA-), or medium with Torin-1 were analysed by means of a m^7^GTP-agarose pulldown assay and western blotting. The phosphorylation status of eIF2α and S6 ribosomal protein in whole-cell lysates (5% input) were determined by staining with specific antibodies. Staining for eIF4E was used as positive control, and for actin as negative controls for m^7^GTP pulldown.

To investigate the role of mTOR signalling in UV-induced translation arrest, we treated U2OS cells with 10 or 20 mJ/cm^2^ UVC light and analysed phosphorylation of the mTOR targets 4E-BP and ribosomal protein S6 by western blotting (Fig. 1A), and protein synthesis rates using a ribopuromycylation assay (Fig. 1B) at 2 h post-UV treatment. We chose this time point for all analyses because even though SG formation is easily detectable at 1 h post-UV exposure (Aulas et al., 2017), we previously determined that SG formation peaks around 2 h post-UV exposure in these cells (Khaperskyy et al., 2014). For a positive control, we used the catalytic inhibitor of mTOR Torin-1 (Thoreen et al., 2009). As expected, Torin-1 treatment decreased phosphorylation of 4E-BP and S6 in both UV-treated and untreated cells, as well as in control sodium arsenite-treated cells. Interestingly, 4E-BP became hyperphosphorylated following UV exposure or arsenite treatments, as indicated by the appearance of phospho-4E-BP bands with reduced electrophoretic mobility (Fig. 1A, compare lane 1 to 3, 5 and 7). UV-induced GCN2 autophosphorylation and GCN2-mediated eIF2α phosphorylation were not affected by mTOR inhibition, indicating that, under the conditions tested, mTOR is not required for GCN2 activation and signalling. The ribopuromycylation assay showed that mTOR inhibition had an additive effect to the UV-induced translation arrest (Fig. 1B,C), and revealed the greater magnitude of translation arrest induced by the higher dose of UV treatment than upon translation inhibition induced by mTOR inhibition (Fig. 1A and B, compare lane 2 to lanes 3 and 5).

Having determined that UV-induced translation arrest is independent of mTOR inhibition, we tested how mTOR inhibition affects UV-induced SG formation. Immunofluorescence microscopy analysis of cells treated with UV light showed that inhibition of mTOR by either Torin-1 or amino acid starvation decreased but did not prevent SG formation (Fig. 1D,E). Inhibition of translation initiation by pharmacological inhibition of mTOR did not induce SG formation in the absence of stress (Fig. 1E). We used amino acid starvation as another control in this experiment, because, unlike with UV light, it is known to cause both GCN2-dependent eIF2α phosphorylation (Harding et al., 2019) and mTOR inhibition (Sancak et al., 2008). SGs did not form in cells incubated in amino acid-free medium without UV exposure, indicating that the eIF2α phosphorylation by GCN2 is not sufficient to drive SG condensation when mTOR is inhibited. Strong inhibition of 4E-BP phosphorylation in Torin-1-treated cells would be expected to cause disassembly of the eIF4F translation preinitiation complex, and our results show that UV-induced SGs can still form in Torin-1-treated cells (Fig. 1E). This suggests that UV-induced SGs can accumulate untranslated mRNPs that lack eIF4F. To determine whether UV damage causes eIF4F complex disassembly that is independent of mTOR inhibition, we compared eIF4F complex formation in UV-treated cells and cells treated with either amino acid-free medium or Torin-1 by performing an m^7^GTP-agarose pulldown assay (Fig. 1F). As expected, mTOR inhibition induced by amino acid starvation or Torin-1 caused dephosphorylation of 4E-BP, its association with eIF4E and inhibited binding of eIF4G to the m^7^GTP beads, reflecting impairment of eIF4E–eIF4G interaction (Fig. 1F, lanes 7 and 8). By contrast, UV treatment did not affect the eIF4E–eIF4G interaction and did not increase the 4E-BP association with eIF4E (Fig. 1F, lane 6). This indicates that SG condensation in response to UV does not correlate with eIF4F complex disassembly nor does it depend on the bulk of untranslated mRNPs, which maintain an intact eIF4F complex (Fig. 1D–F).

### eIF2α phosphorylation is not required for UV-induced SG formation

**Fig. 2.**
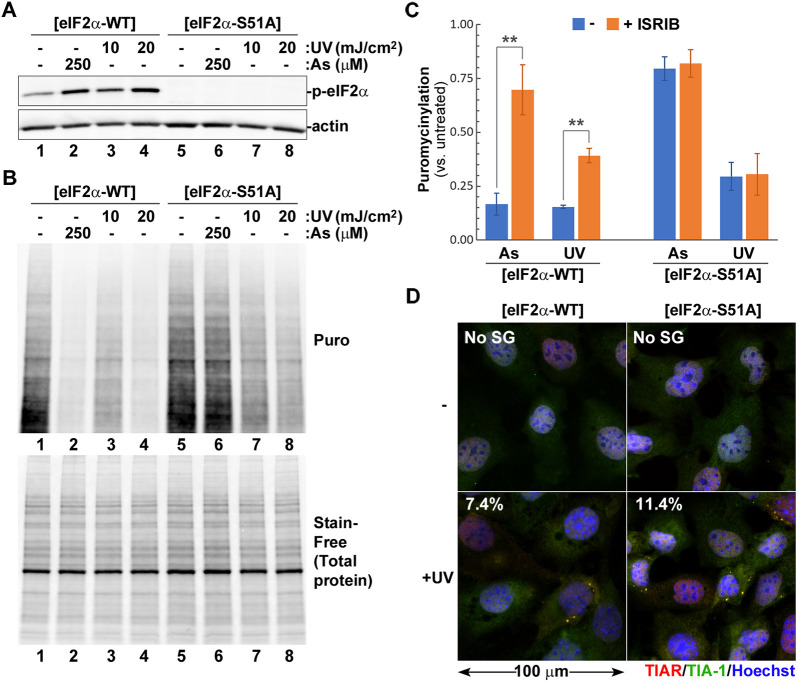
**UV damage triggers phospho-eIF2α-independent translation arrest and SG formation.** UV-induced translation arrest and SG formation were analysed in MEFs engineered to express either wild-type or S51A mutant eIF2α ([eIF2α-WT] or [eIF2α-S51A], respectively). (A) eIF2α phosphorylation in response to sodium arsenite (As) or UV light (UV) were analysed by western blotting. Staining for actin was used as loading control. (B) Protein synthesis rates were analysed in cells treated with sodium arsenite (As) or UV light (UV) using ribopuromycylation assay and western blotting with anti-puromycin antibody (Puro, top panel). Total protein content in each lane was visualised using Stain-Free reagent (bottom panel). (C) Sodium arsenite (As) and UV-induced (UV, 20 mJ/cm^2^) translation arrest was quantified (mean±s.d.) from ribopuromycylation assays of cells incubated with or without ISRIB (*n*=3). **P<0.01 (two-way ANOVA followed by Tukey's multiple comparisons test). (D) SG formation in UV treated (+UV) and control (−) cells was visualised by staining for TIAR (red) and TIA-1 (green). Nuclei were stained with Hoechst dye (blue). Numbers indicate the percentage of cells with SGs quantified from at least three random fields of view containing >100 cells (mean values, *n*=2).

To confirm that eIF2α phosphorylation is not required for SG formation in response to UV damage, we compared translation arrest and SG formation in mouse embryonic fibroblasts (MEFs) engineered to express either the wild-type eIF2α (MEF[eIF2α-WT]) or the unphosphorylatable S51A mutant (MEF[eIF2α-S51A]) (Scheuner et al., 2001). In these experiments we used sodium arsenite as a control, because it is known to induce phospho-eIF2α-dependent translation arrest (McEwen et al., 2005). Treatment of MEF[eIF2α-WT] cells with UV light caused increased eIF2α phosphorylation and decreased protein synthesis rates in a dose-dependent manner, as determined by western blotting and ribopuromycylation assays, respectively (Fig. 2A,B; Fig. S1). In these cells, sodium arsenite triggered eIF2α phosphorylation levels and translation arrest comparable to those induced by 20 mJ/cm^2^ UV (Fig. 2A,B; Fig. S1). As expected, in MEF[eIF2α-S51A] cells no phospho-eIF2α signal was detected by western blotting (Fig. 2A, lanes 5–8), yet a substantial decrease in protein synthesis rates was evident following UV treatment (Fig. 2B; Fig. S1), indicating that UV-induced translation arrest is largely phospho-eIF2α independent. Only a ∼20% translation arrest was induced by sodium arsenite in MEF[eIF2α-S51A] cells (Fig. 2B; Fig. S1), confirming that arsenite inhibits translation via a phospho-eIF2α-dependent mechanism. To gain further insight into the exact contribution of eIF2α phosphorylation to translation inhibition, we carefully quantified puromycin incorporation from three independent experiments comparing treatment with sodium arsenite to exposure to 20 mJ/cm^2^ UV. Experiments were performed as shown in Fig. 2A,B, except that we introduced treatment with the integrated stress response inhibitor (ISRIB) as an additional control to block the effects of eIF2α phosphorylation on translation initiation (Sidrauski et al., 2013, 2015). In MEF[eIF2α-WT] cells, ISRIB increased protein synthesis rates after both arsenite and UV treatment (Fig. 2C). However, even in the presence of ISRIB, UV light caused more than 60% inhibition of translation. By contrast, incubation with ISRIB had no effect on protein synthesis rates in MEF[eIF2α-S51A] cells, as expected, with UV exposure causing over a three times greater inhibition of translation compared to arsenite (Fig. 2C). Next, we analysed UV-induced SG formation in MEF[eIF2α-S51A] cells using immunofluorescence staining, and saw that SGs formed in these cells just as well as in MEF[eIF2α-WT] cells (Fig. 2D). Together, these experiments suggest that, despite strong eIF2α phosphorylation, UV-induced SG formation may be largely driven by phospho-eIF2α-independent translation arrest.

### GCN2 partially contributes to translation arrest and SG formation in response to UV damage

**Fig. 3.**
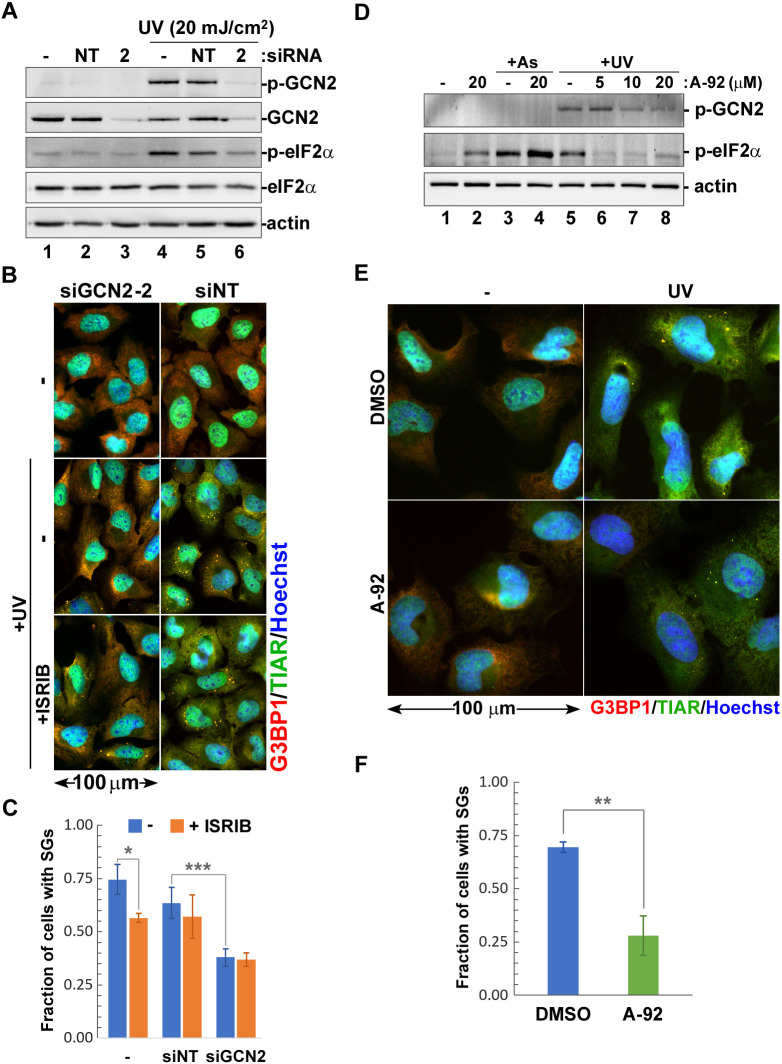
**Catalytic activity of GCN2 is important for SG formation.** (A–C) U2OS cells were transfected with siRNA-2 targeting GCN2 (2), non-targeting siRNA (NT), or left untransfected, and were analysed after 48 h. (A) Effects of GCN2 silencing on eIF2α phosphorylation in response to UV light (UV) were analysed by western blotting. Efficiency of siRNA-mediated knockdown was monitored by staining for total and phosphorylated GCN2. Staining for actin was used as loading control. (B) Immunofluorescence microscopy staining of control or UV-treated (+UV, 20 mJ/cm^2^) cells incubated with or without ISRIB for 2 h post-UV exposure. SG formation was analysed by staining for G3BP1 (red) and TIAR (green). Nuclei were stained with Hoechst dye (blue). (C) UV-induced SG formation was quantified (mean±s.d.) from immunofluorescence microscopy staining represented in panel B (*n*=3). *P<0.05; ***P<0.001 (two-way ANOVA followed by Tukey's multiple comparisons test). (D) Concentration-dependent effects of A-92 on GCN2 and eIF2α phosphorylation in response to 20 mJ/cm^2^ UV light (+UV) were analysed by western blotting. Lysates from untreated cells and cells treated with sodium arsenite (+As) were used as controls. Staining for actin was used as a loading control. (E) Immunofluorescence microscopy staining of control (−) or UV-treated (UV, 20 mJ/cm^2^) cells incubated with 5 µM A-92 or vehicle (DMSO) for 2 h post-exposure. SG formation was visualised by staining for G3BP1 (red) and TIAR (green). Nuclei were stained with Hoechst dye (blue). (F) SG formation in control or A-92 treated cells was quantified (mean±s.d.) from immunofluorescence microscopy staining (*n*=3) of cells exposed to 20 mJ/cm^2^ UV. **P<0.01 (two-tailed Student's *t*-test).

To examine how GCN2 contributes to UV-induced translation arrest and SG formation, we compared these responses in control U2OS cells, cells transfected with non-targeting siRNA, and cells in which GCN2 expression was silenced using transfection with specific siRNAs (Fig. 3A–C; Fig. S2). Western blotting analysis of whole-cell lysates at 48 h post-transfection demonstrated that GCN2-specific siRNAs caused significant depletion of total GCN2 protein compared to untransfected cells or cells transfected with control non-targeting siRNA (Fig. 3A; Fig. S2). Importantly, siRNA-mediated knockdown of GCN2 resulted in diminished eIF2α phosphorylation in response to UV treatment (Fig. 3A, lane 6). A ribopuromycylation assay revealed that, in cells transfected with one of the two siRNAs (siGCN2-1), the basal rates of protein synthesis were decreased compared to cells transfected with either siGCN2-2 or non-targeting siRNA control (Fig. S2B, compare lane 3 to lanes 2 and 4). This effect, however, was likely to be caused by unknown off-target effects, as the degree of GCN2 silencing was comparable between two siRNAs (Fig. S2A, lanes 3, 4, 7 and 8). When we quantified rates of protein synthesis in control and GCN2-silenced cells following UV treatment, we observed that GCN2 knockdown partially restored translation (Fig. S2C). Interestingly, in cells transfected with either GCN2-specific siRNA, the magnitude of UV-induced translation arrest was not affected by ISRIB treatment and was generally comparable to the magnitude of UV-induced translation arrest in control cells treated with ISRIB. This suggests that the difference in translation arrest between control and GCN2 knockdown cells can be largely attributed to the effects of GCN2-mediated eIF2α phosphorylation. Next, we analysed UV-induced SG formation in control and GCN2 knockdown cells by immunofluorescence staining (Fig. 3B) and quantified SG formation from three independent experiments (Fig. 3C). Our analyses revealed that GCN2 silencing decreased SG formation. ISRIB treatment had no effect on SG formation, except for a slight decrease in untransfected cells, which was statistically significant. Given the profound effects of siGCN2-1 on basal protein synthesis rates in untreated cells, we compared the effects of siGCN2-1 and siGCN2-2 on arsenite-induced SG formation. Sodium arsenite induces SG formation independently from GCN2 through eIF2α phosphorylation by HRI (McEwen et al., 2005), yet siGCN2-1 transfection severely affected SG formation following arsenite treatment (Fig. S2E). This indicates that siGCN2-1 has strong off-target effects that interfere with SG formation in general and cannot be used for analyses of GCN2-mediated effects specifically. Nevertheless, given that siGCN2-2 inhibited SG formation in response to UV but not sodium arsenite, our results suggest that GCN2 contributes to the efficiency of SG formation caused by UV damage.

### Catalytic activity of GCN2 enhances UV-induced SG formation

Our analysis of the contributions of GCN2 to translation arrest and SG formation in response to UV damage using siRNA silencing revealed that GCN2-mediated phosphorylation of eIF2α is responsible for approximately one-third of the translation inhibition following UV exposure (Fig. S2C). Interestingly, while eIF2α phosphorylation did not contribute to SG formation, silencing of GCN2 consistently decreased SG formation by at least 30% (Fig. 3C). To determine whether GCN2 catalytic activity is important for UV-induced SG formation, we analysed formation of SGs following 20 mJ/cm^2^ UV exposure in cells treated with GCN2 inhibitor A-92 (also known as GCN2-IN-1) and in control DMSO-treated cells (Fig. 3D–F). First, we established the optimal concentration of A-92 by analysing eIF2α phosphorylation in response to UV light by western blotting (Fig. 3D). Unexpectedly, the highest concentration tested (20 µM A-92) caused increased phospho-eIF2α even in untreated cells (Fig. 3D, compare lanes 1 and 2), possibly due to off-target cytotoxicity. For analyses of SG formation, we used 5 µM A-92 treatment because it was sufficient for preventing eIF2α phosphorylation without major effects on the upstream activation and autophosphorylation of GCN2 itself (Fig. 3D, compare lanes 6, 7, and 8). Immunofluorescence staining of cells at 2 h post-UV treatment revealed that compared to DMSO control, A-92 caused formation of SGs in fewer cells (Fig. 3E). Quantification of the proportion of SG-positive cells showed that there was significant decrease in the presence of A-92, confirming that the catalytic activity of GCN2 is important for SG formation in response to UV damage (Fig. 3F). Since UV-induced SG formation is phospho-eIF2α independent, our data points to a potential involvement of other as yet unidentified GCN2 targets.

### GCN2 is required for UV-induced SG formation in interphase cells

**Fig. 4.**
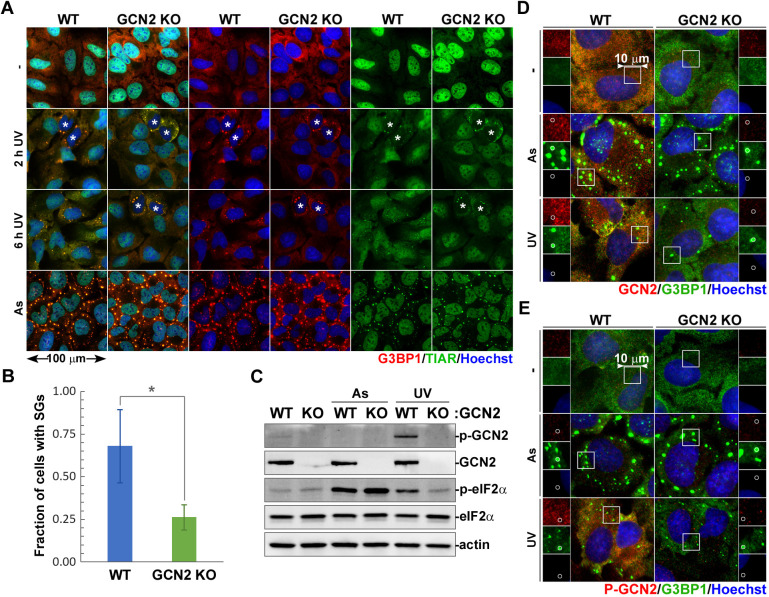
**UV-induced SG formation is decreased in GCN2-deficient cells.** SG formation was compared in parental U2OS cells (WT) and GCN2-knockout cells generated using CRISPR/Cas9 (GCN2 KO). (A) SG formation was analysed by immunofluorescence microscopy in cells exposed to 20 mJ/cm^2^ UV light at 2 and 6 h post-exposure (UV), untreated control cells and cells treated sodium arsenite (As), and stained for G3BP1 (red) and TIAR (green). Nuclei were stained with Hoechst dye (blue). Asterisks denote cells that underwent mitosis within the time frame of the experiment (pairs of cells with predominantly cytoplasmic TIAR). (B) UV-induced SG formation in parental U2OS cells and GCN2 knockout cells was quantified at 2 h post-UV exposure from immunofluorescence microscopy staining (mean±s.d.; *n*=3). *P<0.05 (two-tailed Student's *t*-test). (C) Lack of GCN2 expression in knockout cells and its effect on eIF2α phosphorylation in response to UV light (UV) or sodium arsenite (As) were analysed by western blotting. Staining for actin was used as loading control. (D) Immunofluorescence microscopy of control (−), UV-treated (UV, 20 mJ/cm^2^), or arsenite-treated (As) cells stained for total GCN2 (red) and G3BP1 (green). Nuclei were stained with Hoechst dye (blue). (E) Immunofluorescence microscopy of cells treated as in D and stained for phospho-GCN2 (red) and G3BP1 (green). Nuclei were stained with Hoechst dye (blue).

Our analyses using siRNA silencing and chemical inhibition of GCN2 revealed that this kinase enhances SG formation in UV-treated cells; however, many cells still formed SGs when GCN2 activity was inhibited. One explanation for this phenotype could be that neither siRNA knockdown nor A-92 were able to completely abrogate GCN2 activity and some residual GCN2 function contributed to SG formation. Alternatively, GCN2 may be affecting SG nucleation dynamics or it could be required for SG formation only in a certain subpopulation of cells. To distinguish between these possibilities, we generated GCN2-knockout U2OS cells using CRISPR/Cas9 and compared UV-induced SG formation in these and parental U2OS cells at 2 h and 6 h post-exposure (Fig. 4A). Lack of GCN2 did not affect SG formation in control sodium arsenite-treated cells and did not abrogate UV-induced SG formation in some cells (Fig. 4A–C). At the same time, we noticed that those GCN2-knockout cells that formed SGs were usually found in pairs and had distinctively cytoplasmic TIAR staining (Fig. 4A). The majority of cells remained SG-free even at the later 6 h timepoint, while more than half of all the parental U2OS cells formed SGs (Fig. 4A). Neither phosphorylated nor total GCN2 accumulated in stress granules (Fig. 4D,E). The distinct morphology of SG-positive GCN2-deficient cells that were found in pairs suggested that these cells have undergone division and have not imported the bulk of TIAR into the newly formed nuclei. UV damage is well known to induce prolonged cell cycle arrest (Moutaoufik et al., 2014), and most likely these cells were committed to mitosis prior to UV exposure and were irradiated right before, during, or immediately after division. To test this hypothesis, we visualised UV-induced SG formation in live A549 cells stably expressing the EGFP–G3BP1 reporter with or without treatment with GCN2 inhibitor A-92 starting from 1 h prior to UV exposure (Fig. S3). As a control, we used sodium arsenite treatment, which triggered SG formation peaking at 1 h and was followed by dissolution in most cells by 3 h and then cell rounding and death at 6 h (Fig. S3A,C). UV-irradiated control DMSO-treated cells started forming SGs as early as 30 min post-UV exposure, and numbers of SG-positive cells continued to increase throughout the 6-h time course (Fig. S3C). Most notably, cells that divided during the course of the experiment formed SGs earlier than the interphase cells (Fig. S3B). By contrast, number of SG-positive cells treated with the GCN2 inhibitor A-92 plateaued at 3 h post-UV exposure (Fig. S3C) and only cells that underwent division immediately prior to or at the time of UV exposure formed SGs (Fig. S3B). Taken together, our analysis of fixed and live cells indicates that the catalytic activity of GCN2 is required for UV-induced SG formation in interphase cells, and that SG formation in cells irradiated during mitosis is GCN2-independent.

### UV-induced SG condensation requires G3BP1 but not G3BP2

**Fig. 5.**
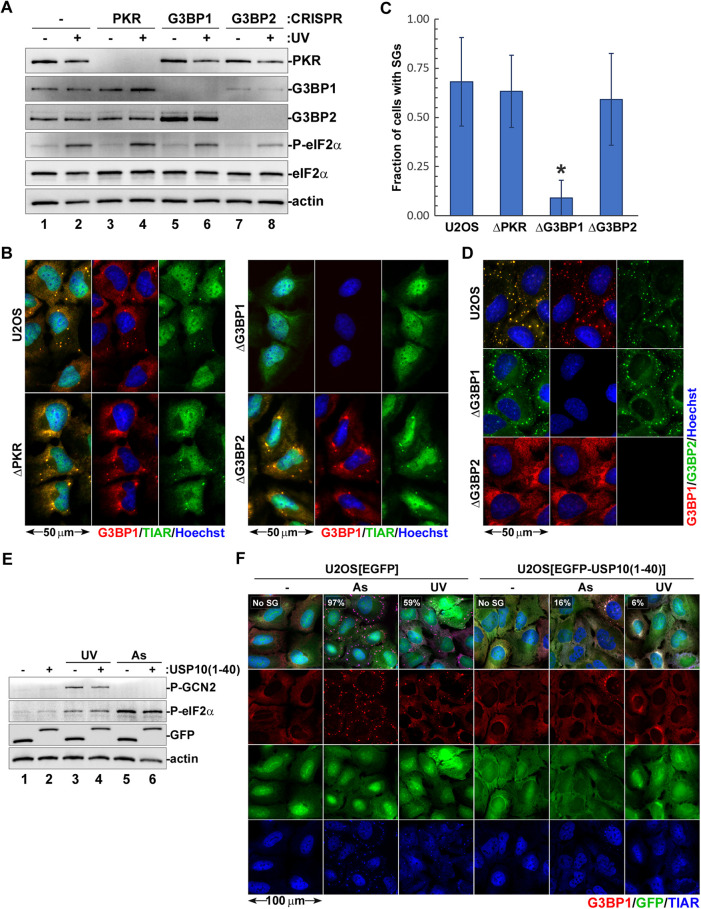
**UV-induced SG formation is dependent on G3BP1.** (A–D) G3BP1, G3BP2, and control PKR knockout cells were generated using CRISPR/Cas9. (A) PKR, G3BP1 and G3BP2 expression in CRISPR knockout cells and eIF2α phosphorylation in response to 20 mJ/cm^2^ UV light (UV) were analysed by western blotting. Staining for actin was used as loading control. (B) UV-induced SG formation (20 mJ/cm^2^) was analysed by immunofluorescence microscopy in parental U2OS cells (U2OS) and CRISPR knockout cell lines lacking G3BP1 (ΔG3BP1), G3BP2 (ΔG3BP2) or PKR (ΔPKR), and stained for G3BP1 (red) and TIAR (green). Nuclei were stained with Hoechst dye (blue). (C) UV-induced SG formation was quantified (mean±s.d.) from immunofluorescence microscopy staining represented in B (*n*=3). *P<0.05 (two-way ANOVA followed by Tukey's multiple comparisons test). (D) Selenite-induced SG formation was analysed by immunofluorescence microscopy in parental U2OS cells (U2OS) and CRISPR knockout cell lines lacking G3BP1 (ΔG3BP1) or G3BP2 (ΔG3BP2) stained for G3BP1 (red) and G3BP2 (green). Nuclei were stained with Hoechst dye (blue). (E,F) Expression of the EGFP–USP10(1-40) fusion protein or EGFP control was induced in U2OS cells stably transduced with lentiviral constructs encoding these proteins. After 48 h, cells were left untreated (−), or were treated with UV light (UV, 20 mJ/cm^2^) or sodium arsenite (As). (E) Phosphorylation status of GCN2 and eIF2α post-treatment was analysed by western blotting, as well as the expression of EGFP–USP10(1-40) fusion protein or EGFP control. Staining for actin was used as loading control. (F) SG formation was analysed by immunofluorescence microscopy staining for G3BP1 (red) and TIAR (blue) in these GFP-positive cells (green). Numbers indicate the percentage of cells with SGs quantified from at least three random fields of view containing >100 cells (mean values, *n*=2).

Different types of SGs require different nucleating proteins, with G3BP1 and/or G3BP2 being necessary for SG formation in response to variety of stresses, with just a few notable exceptions (Kedersha et al., 2016). To test whether UV-induced SGs require G3BP1 and/or G3BP2 for their formation, we generated G3BP1 and G3BP2 knockout U2OS cells and control PKR eIF2α kinase knockout cells using CRISPR/Cas-9. PKR is not activated by UV light (Deng et al., 2002) and, as expected, UV-induced SG formation was not affected in PKR-deficient cells (Fig. 5A–C). Similarly, SG formation following UV exposure was not affected in G3BP2-deficient cells (Fig. 5B), even though G3BP1 expression was also reduced in these cells (Fig. 5A). By contrast, G3BP1 knockout strongly inhibited UV-induced SG formation (Fig. 5B,C). As observed previously by Kedersha et al. (2016), G3BP1 disruption resulted in upregulation of G3BP2 expression (Fig. 5A); however, it did not compensate for the lack of G3BP1. Conversely, G3BP1-negative cells formed SGs following treatment with sodium selenite, while lack of G3BP2 blocked selenite-induced SG formation (Fig. 5D). Thus, our analysis reveals that UV-induced SGs require G3BP1, but not G3BP2, for their formation. Even when G3BP2 expression is increased in G3BP1-deficient cells (Fig. 5A), this protein cannot nucleate UV-induced SG condensation. In the presence of G3BP1, however, G3BP2 is recruited to SGs triggered by UV exposure (Fig. S4A).

G3BP1-dependent SG condensation requires interaction of its NTF-2-like domain with caprin-1 protein, and this interaction can be disrupted by the N-terminal 40-amino-acid long region of USP10 (Kedersha et al., 2016; Solomon et al., 2007). To test whether a caprin-1-dependent mechanism of SG condensation is required for UV-induced SG formation, we generated U2OS cells carrying doxycycline-inducible EGFP fused to the N-terminal 40-amino-acid peptide of USP10 {U2OS[iEGFP-USP10(1-40)]} and the control U2OS[iEGFP] cells. Following induction of EGFP–USP10(1-40) or control EGFP protein expression, we treated these cells with UV or sodium arsenite, and analysed GCN2 and eIF2α phosphorylation using western blotting (Fig. 5E) and SG formation using immunofluorescence staining (Fig. 5F). As expected, the eIF2α phosphorylation in response to UV or sodium arsenite was not affected by fusion protein expression (Fig. 5E). By contrast, SG formation in response to both treatments was blocked in EGFP-USP10(1-40)-expressing cells (Fig. 5F). Thus, UV-induced SG condensation requires G3BP1 and can be blocked by disrupting interactions of its NTF-2-like domain.

### UV-induced SGs do not accumulate eIF4G and eIF3B

**Fig. 6.**
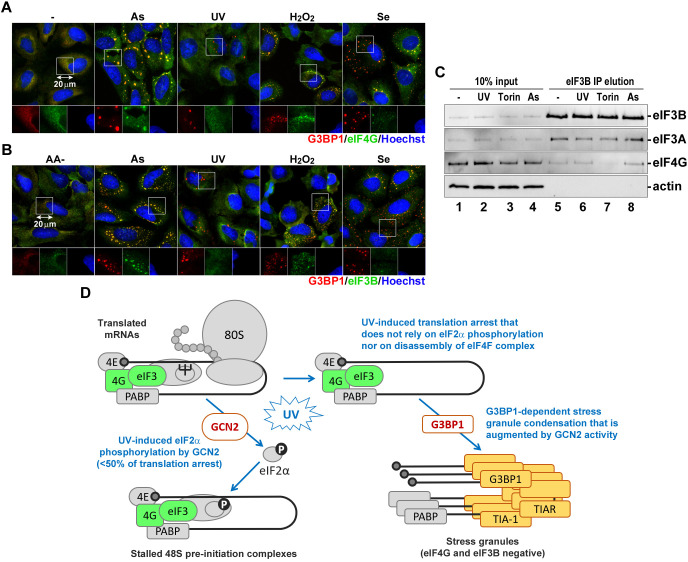
**Ribonucleoproteins recruited to UV-induced SGs selectively exclude eIF4G and eIF3B.** (A) Cells treated with sodium arsenite (As), UV light (UV, 20 mJ/cm^2^), H_2_O_2_ or sodium selenite (Se) were analysed by immunofluorescence staining for the SG markers G3BP1 (red) and eIF4G (green). Nuclei were stained with Hoechst dye (blue). (B) Cells incubated in amino acid-free medium (AA-) or treated with sodium arsenite (As), UV light (UV, 20 mJ/cm^2^), H_2_O_2_ or sodium selenite (Se) were analysed by immunofluorescence staining for the SG markers G3BP1 (red) and eIF3B (green). Nuclei were stained with Hoechst dye (blue). (C) Association of the eIF3 complex with eIF4G was analysed using co-immunoprecipitation from cytoplasmic lysates of untreated control cells (−) or cells treated with UV light (UV, 20 mJ/cm^2^), Torin-1 (Torin) or sodium arsenite (As). Presence of eIF3A and eIF4G proteins in cytoplasmic lysates (10% input) and eIF3B co-immunoprecipitation samples (IP elution) was analysed by western blotting. Staining for eIF3B was used as positive control, and for actin as negative control. (D) Schematic diagram depicting the working model for UV-induced translation arrest and SG formation. GCN2-mediated eIF2α phosphorylation only partially contributes to overall translation arrest; however, most untranslated mRNPs remain associated with eIF4G and eIF3. SGs form in a G3BP1-dependent manner but recruit only a small fraction of untranslated mRNAs and exclude eIF4G and eIF3.

All our results so far suggest that UV damage induces G3BP1-dependent SG formation that does not require mTOR inhibition or eIF2α phosphorylation – two main pathways that can lead to the influx of untranslated mRNPs in the cytoplasm of cells under stress. Previous studies have indicated that UV-induced SGs lack eIF4G and eIF3B (Aulas et al., 2017). These proteins are associated with stalled 48S pre-initiation complexes that accumulate following translation arrest induced by eIF2α phosphorylation (Kedersha et al., 2002). To confirm that UV-induced SGs indeed lack eIF4G and eIF3B in our system, we analysed SG composition using immunofluorescence microscopy. As controls, we used treatments with sodium arsenite, H_2_O_2_ and sodium selenite. The latter two treatments were shown to induce SGs that do not accumulate eIF3B (Emara et al., 2012; Fujimura et al., 2012). Interestingly, in our system, both eIF4G and eIF3B were recruited to some SGs formed in response to H_2_O_2_ and sodium selenite (Fig. 6A,B), which may indicate slight differences in experimental conditions and/or specificity of antibodies used in our study. By contrast, our analysis revealed that UV-induced SGs did not recruit eIF4G or eIF3B in U2OS cells (Fig. 6A,B), indicating that despite the majority of mRNPs maintaining an interaction with eIF4G following UV exposure (Fig. 1E), they do not represent the main constituents of SGs in our system. In agreement with this model, we could not detect accumulation of PABP, 4E-BP or ribosomal protein S6 in UV-induced SGs (Fig. S4), while HuR (also known as ELAVL1), another common SG marker that binds 3′ untranslated regions of mRNAs, was recruited to both arsenite and UV-induced SGs (Fig. S4B).

Next, to determine whether eIF4G-eIF3B interactions are disrupted after UV treatment of U2OS cells, we conducted co-immunoprecipitation assays using the same anti-eIF3B antibody that we used for immunofluorescence staining in Fig. 6B. Torin-1 served as a positive control, whereas sodium arsenite served as a negative control that does not disrupt integrity of 48S pre-initiation complexes. As expected, in untreated and sodium arsenite-treated cells, similar amounts of eIF4G co-immunoprecipitated with eIF3B (Fig. 6C, lanes 5 and 8). In Torin-1-treated cells, eIF4G did not co-immunoprecipitate with eIF3B (Fig. 6C, lane 7). In UV-treated cells, the eIF4G–eIF3B interaction was not disrupted and amounts of co-immunoprecipitated eIF4G were similar to those in untreated or sodium arsenite-treated cells (Fig. 6C, lane 6). It is important to note that under all conditions tested, eIF3A, another subunit of eIF3 core complex, co-immunoprecipitated efficiently with eIF3B (Fig. 6C). Thus, our data is consistent with the working model where most 48S pre-initiation complexes remain intact after UV damage, similar to those that accumulate in cells treated with sodium arsenite. At the same time, UV-induced SGs form via a G3BP1-dependent mechanism but accumulate only a fraction of untranslated mRNPs, which lack eIF4G and eIF3B (Fig. 6D).

## DISCUSSION

Depending on the type, magnitude or duration of stress, SG formation has been linked with cell survival, apoptosis, regulation of the cell cycle and innate immune signalling (Aulas et al., 2018; Kim et al., 2005; Moutaoufik et al., 2014; Reineke and Lloyd, 2015). Nevertheless, the exact molecular functions of these condensates remain poorly understood. Studies into mechanisms of SG formation and functions are complicated by the fact that various types of stress trigger formation of SGs that differ significantly in their properties and composition (Aulas et al., 2017). In this work, we focused on the mechanisms of translation arrest and SG formation triggered by UV damage. When cells are exposed to various doses of UV, they arrest the cell cycle until the DNA damage induced by UV light can be repaired (Batista et al., 2009). High-dose UV exposure also causes translation arrest and SG formation (Aulas et al., 2017; Burgess et al., 2011; Deng et al., 2002; Moutaoufik et al., 2014). In cultured mammalian cells, doses of 1 mJ/cm^2^ and above are sufficient for cell cycle arrest (Cadet and Douki, 2018); however, doses as high as 5 mJ/cm^2^ are required for induction of eIF2α phosphorylation and inhibition of protein synthesis, and up to 20 mJ/cm^2^ is required for reliable SG induction at 1–2 h post-exposure (Aulas et al., 2017; Kedersha and Anderson, 2007). Since our analyses were focused specifically on SG formation and similar doses were used by others previously, we decided to use doses of 10–20 mJ/cm^2^ in our experiments.

The ‘canonical’ stress granules form as part of the ISR and their formation is initiated by phosphorylation of eIF2α by one of the four kinases that become activated by different types of stress. The classical example of canonical SGs are those induced by treatment with sodium arsenite – the most robust and widely used SG inducer, which triggers eIF2α phosphorylation by HRI (McEwen et al., 2005). Exposure to UV light causes eIF2α phosphorylation by a different kinase, GCN2 (Deng et al., 2002). However, previous studies using eIF2α-S51A mutant human HAP1 cells or MEF[eIF2α-S51A] cells have demonstrated that UV-induced SG formation is phospho-eIF2α independent (Aulas et al., 2017; Moutaoufik et al., 2014). Therefore, we tested whether UV inhibits translation initiation and induces SG formation by causing inhibition of mTOR and disassembly of the eIF4F complex. Our analysis of the phosphorylation status of mTOR targets 4E-BP and S6 by western blotting and the assembly of eIF4F complex using an m^7^GTP-agarose pulldown assay revealed that mTOR activity and eIF4F complex formation remained unaffected (Fig. 1A,F). Importantly, Torin-1 treatment did not enhance but instead inhibited SG formation triggered by UV light, providing additional evidence that UV-induced SG formation is not driven by mTOR inhibition (Fig. 1D,E). One possible explanation for decreased SG formation after mTOR inhibition is induction of autophagy – the catabolic process proposed to accelerate SG disassembly (Monahan et al., 2016; Protter and Parker, 2016). Alternatively, SG formation following UV damage may require initial influx of intact 48S translation preinitiation complexes that become modified as part of the mechanism of SG condensation.

Torin-1 directly inhibits the catalytic activity of mTOR and does not activate ISR or induce SG formation in the absence of stress, even though it causes translation inhibition and polysome disassembly (Thoreen et al., 2009). By contrast, amino acid starvation simultaneously causes the inhibition of mTOR activity and accumulation of uncharged tRNAs that activate GCN2 and trigger GCN2-mediated eIF2α phosphorylation (Sancak et al., 2008; Wek et al., 1995). SG formation can be induced in HeLa cells following 2 h of amino acid starvation (Damgaard and Lykke-Andersen, 2011). However, similar to what occurs after Torin-1 treatment, amino acid deprivation alone did not trigger SG formation in U2OS cells in our system (Fig. 1E). This may be due to differences in response to amino acid starvation between HeLa and U2OS cells or small variations in the exact composition of the amino acid-free medium between our study and the study by Damgaard and Lykke-Andersen (2011); nevertheless, our results clearly show that translation arrest triggered by amino acid starvation and UV light have different mechanisms despite causing eIF2α phosphorylation through the same kinase, GCN2.

Previous studies have shown that eIF2α phosphorylation is not required for UV-induced SG formation. At the same time, cells engineered to express unphosphorylatable S51A eIF2α mutant are more susceptible to UV damage-induced cell death (Aulas et al., 2017; Burgess et al., 2011). Therefore, we quantified the contribution of eIF2α phosphorylation to the magnitude of translation arrest following UV exposure. In MEFs expressing a non-phosphorylatable eIF2α (MEF[eIF2α-S51A]), UV treatment inhibited translation; however, the magnitude of UV-induced translation arrest measured by ribopuromycylation assay was lower compared to that of the wild-type MEFs (Fig. 2A–C). As expected, translation arrest in MEF[eIF2α-S51A] cells was not affected by treatment with ISRIB, which acts by negating effects of eIF2α phosphorylation on translation initiation. Our results indicate that even though the UV-induced translation arrest mechanism does not act exclusively through eIF2α phosphorylation, it does contribute to its magnitude. At the same time, we confirmed previous reports that UV-induced SG formation is independent of eIF2α phosphorylation (Fig. 2D) (Aulas et al., 2017; Moutaoufik et al., 2014). Apart from inhibiting bulk protein synthesis, eIF2α phosphorylation allows for preferential synthesis of proteins involved in ISR (Jackson et al., 2010). The best-known example of mRNAs that become translated more efficiently is the mRNA encoding activating transcription factor 4 (ATF4). In its 5′ region, it contains short upstream open reading frames (uORFs) that inhibit initiation of protein synthesis from the downstream ATF4 ORF under normal conditions. When initiation is inhibited by stress-induced eIF2α phosphorylation, the downstream ORF becomes more accessible and expression of ATF4 is induced (Harding et al., 2000b). This transcription factor then translocates to the nucleus where it activates the stress response gene expression programme (Harding et al., 2003). Given that eIF2α phosphorylation is not required for UV-induced translation arrest and SG formation, yet plays an important role in cell survival, it is tempting to speculate that its role in regulating synthesis of ISR genes like ATF4 is contributing to a better stress response. However, it was demonstrated previously that, due to inhibition of transcription caused by DNA damage, UV exposure does not upregulate ATF4 protein levels (Dey et al., 2010). Therefore, the exact reason for decreased survival of cells expressing unphosphorylatable eIF2α remains unknown.

Having examined the contribution of eIF2α phosphorylation to the magnitude of translation arrest in response to UV damage, next we examined the role of the eIF2α kinase GCN2. In addition to UV light, this kinase can be activated by other stress stimuli, including amino acid starvation, inhibition of the ubiquitin proteasome system by MG132 treatment and even viral infections (Berlanga et al., 2006; Deng et al., 2002; Mazroui et al., 2007; Wek et al., 1995). The mechanism of GCN2 activation is best understood for amino acid starvation, which leads to accumulation of deacylated tRNAs. GCN2 possesses a histidyl-tRNA synthetase-like (HisRS) domain close to the C-terminus that is involved in autoinhibitory interactions in the inactive GCN2 dimer. Binding of ‘uncharged’ deacylated tRNAs by the HisRS domain causes structural rearrangements in the GCN2 dimer interface allowing for autophosphorylation and activation of the kinase (Masson, 2019; Wek et al., 1995). Another tRNA-independent mechanism of GCN2 activation mediated by the P-stalk of ribosomes stalled during elongation has been described, which can complement tRNA-dependent activation when amino acids are depleted (Harding et al., 2019; Inglis et al., 2019). How GCN2 is activated by UV light and whether it involves ribosomal stalling on UV-damaged mRNAs is not known, but regardless of the triggering stimuli, the main target of the activated kinase is eIF2α. How many other GCN2 phosphorylation targets exist is presently unknown; however, it has been shown that it can phosphorylate at least one additional protein, methionyl-tRNA synthetase (MRS; also known as MARS1) (Kwon et al., 2011). The GCN2-mediated phosphorylation of MRS is directly involved in the UV damage response because it causes dissociation of MRS and the aminoacyl-tRNA synthetase-interacting multifunctional protein-3 (AIMP3). The latter then translocates to the nucleus where it participates in the DNA damage response. Another consequence of MRS phosphorylation is the inhibition of methionyl-tRNA generation and depletion of the eIF2–GTP–Met-tRNA^Met^ ternary complex, which is required for translation initiation, but through a mechanism independent of eIF2α phosphorylation (Kwon et al., 2011). This could potentially explain how UV-induced translation arrest can be largely eIF2α-independent but still require GCN2. However, when we silenced GCN2 expression in U2OS cells using siRNAs, the translation arrest after UV exposure was greatly reduced compared to control cells but still evident (Fig. S2B,C). These results, together with MEF data (Fig. 2C), further support the idea that a GCN2-mediated eIF2α phosphorylation contributes significantly to the magnitude of UV-induced translation arrest, but there is an additional eIF2α-independent effect of GCN2. By contrast, silencing or genetic deletion of GCN2 significantly decreased UV-induced SG formation (Figs 3C and 4B), indicating that it may be involved, directly or indirectly, in the mechanism of SG nucleation. Further analysis using a specific inhibitor of GCN2, A-92, showed that the catalytic activity of the GCN2 contributes to UV-induced SG formation mechanism (Fig. 3F), especially in interphase cells (Fig. S3B). Thus, the catalytic activity of GCN2 is important for UV-induced SG formation, but not because of the eIF2α phosphorylation or other direct effects on translation. By contrast, SG formation in cells irradiated during mitosis was independent of GCN2 (Fig. 4A; Fig. S3B). In future it will be important to analyse how GCN2-dependent SG formation affects stress responses and cell cycle arrest following UV damage and delineate more precisely which phases of the cell cycle require GCN2 activity for SG nucleation.

Our analysis of UV-induced SG formation revealed that these granules differ from both canonical SGs that require eIF2α phosphorylation and the 4E-BP-dependent SGs that form upon stress-induced inhibition of mTOR. Next, we tested whether UV-induced SG condensation requires G3BP1/2 proteins. Depending on the type of stress, G3BP1 and G3BP2, which share 70% amino acid sequence similarity, can only partially substitute for each other during SG formation (Kedersha et al., 2016). In U2OS cells, silencing of either protein can cause compensatory increases in the levels of the other; however, disruption of G3BP1 or G3BP2 expression individually still decreased SG formation induced by sodium arsenite. By contrast, formation of SGs induced by clotrimazole treatment is impaired only when both G3BP genes are silenced simultaneously, while osmotic or heat shock cause SG formation by a mechanism that is independent of both G3BP1 and G3BP2 (Kedersha et al., 2016). In our study, we used CRISPR/Cas9-mediated disruption of G3BP1 or G3BP2 genes in U2OS cells independently, and compared the contribution of these proteins to UV-induced SG formation. In parallel, we tested the effects of G3BP1 and G3BP2 on SG induction by sodium selenite. In our system, disruption of G3BP1 expression prevented UV-induced SG formation but had no effect on SG formation triggered by selenite (Fig. 5B–D). By contrast, UV induced SGs in G3BP2-deficient cells; however, G3BP2 was required for selenite-induced SG formation (Fig. 5D). Future studies should reveal what differences between G3BP1 and G3BP2 are responsible for selective requirements of these proteins for UV-induced vs selenite-induced SG formation in U2OS cells, but the reliance on G3BP1 suggests that UV-induced SGs can form via the previously established mechanism that requires binding of caprin-1 protein to the NTF2 domain of G3BP1 (Kedersha et al., 2016). This mechanism of SG formation can be inhibited by ectopic overexpression of the N-terminal peptide of USP10, which contains a conserved FGDF sequence motif. This motif is responsible for competitive binding of USP10 to the NTF2 domain of G3BP1 and displacement of caprin-1 (Kedersha et al., 2016). Similarly, several viral proteins that contain the FGDF motif can inhibit SG formation in infected cells (Panas et al., 2015a). Consistent with the Caprin-1-dependent mechanism of SG formation, when we overexpressed the N-terminal 40-amino-acid peptide of USP10 fused to the EGFP in U2OS cells, UV-induced SG formation was blocked (Fig. 5F). These results convincingly show that untranslated mRNPs that form UV-induced SGs phase separate via a mechanism similar to that of canonical sodium arsenite-induced SGs and requires G3BP1 NTF2 domain interactions.

Having determined that UV-induced SG formation in our system is driven by G3BP1 and is not associated with disassembly of the eIF4F complex, we wanted to confirm that the SGs that we detect in U2OS cells at 2 h post-exposure to UV light do not accumulate eIF4G and eIF3B, as was reported by others in different cell types (Aulas et al., 2017; Moutaoufik et al., 2014). As shown in Fig. 6, unlike SGs that formed in response to sodium arsenite, UV-induced SGs did not recruit eIF4G or eIF3B in our system. Interestingly, while we could detect eIF3B in some SGs induced by the H_2_O_2_ or sodium selenite, even the largest foci in UV-treated cells lacked eIF3B signal (Fig. 6B). To rule out the possibility that UV causes selective dissociation of eIF3 complex from eIF4F, we performed co-immunoprecipitation using eIF3B-specific antibody and analysed the eIF3B–eIF4G interaction (Fig. 6C). Similar to what was seen upon sodium arsenite treatment, UV exposure did not disrupt the eIF3B–eIF4G interaction. Together with our analysis of eIF4F complex formation using m^7^GTP-agarose pulldown (Fig. 1F), these results indicate that UV-induced translation arrest acts downstream of 48S complex assembly, partially through GCN2-mediated eIF2α phosphorylation and partially through a yet to be identified GCN2- and eIF2α-independent mechanism. At the same time, UV-induced SG formation mechanism acts on a subset of mRNPs that lose their association with eIF4G and eIF3 as an additional step downstream of the eIF2α-independent translation arrest (Fig. 6D).

## MATERIALS AND METHODS

### Cells and treatments

U2OS human osteosarcoma cells were purchased from American Type Culture Collection (ATCC, Manassas, VA, USA). The eIF2α knock-in mouse embryonic fibroblasts (wild-type and S51A mutant) were a kind gift from Dr Randal Kaufman (Sanford Burnham Prebys Medical Discovery Institute, La Jolla, CA, USA) (Scheuner et al., 2001). A549 cells stably expressing EGFP–G3BP1 fusion protein are described in Khaperskyy et al. (2012). All cell lines were routinely tested for mycoplasma contamination and used at passage <10 after removal from cryostorage. Unless specified otherwise, reagents were purchased from Thermo Fisher Scientific (Waltham, MA). All cell lines were cultured in Dulbecco's modified Eagle's medium (DMEM) supplemented with 10% fetal bovine serum and 2 mM L-glutamine at 37°C in a 5% CO_2_ atmosphere.

For UV light exposure, cells were grown to 65–85% confluency, medium was removed, monolayers washed briefly with phosphate-buffered saline (PBS) and exposed to 10 or 20 mJ/cm^2^ UV light (254 nm, UVC) in a HL-2000 Hybrilinker chamber (UVP), promptly overlaid with fresh warm medium and returned to a 37°C incubator.

For amino acid deprivation experiments, the growth medium was replaced with Hanks' balanced salt solution (HBSS) supplemented with 10% dialysed fetal bovine serum (Wisent Inc., St-Bruno, QC, Canada). For ISRIB or GCN2 inhibitor treatments, the growth medium was replaced with fresh medium containing 200 nM ISRIB (MilliporeSigma, Burlington, MA, USA) or the indicated concentrations of A-92 (also known as GCN2-IN-1; MedChemExpress, Monmouth Junction, NJ, USA). Other treatments were performed by direct addition of a 1:100 volume of stocks pre-diluted in medium. Final concentrations were as follows: 250 nM Torin-1 (TOCRIS, Oakville, ON, Canada); 500 µM sodium arsenite (unless specified otherwise); 1 mM sodium selenite; and 1 mM H_2_O_2_ (all MilliporeSigma, Burlington, MA, USA).

### Generation of inducible cell lines expressing the USP10 N-terminal 40-amino-acid peptide

Generation of cell lines harbouring doxycycline-inducible protein expression constructs using lentiviral vectors based on the pTRIPZ plasmid (Thermo Fisher Scientific) was previously described in Khaperskyy et al. (2016). To enable tight control of the USP10 N-terminal 40-amino-acid peptide expression, it was cloned as an EGFP fusion protein into pTRIPZ vector downstream of doxycycline-inducible promoter between AgeI and MluI sites, completely substituting the turboRFP and shRNA cassette (the MluI site was destroyed during cloning), producing pTRIPZ-EGFP-USP10(1-40) vector (full sequence is available upon request). A companion control plasmid pTRIPZ-EGFP containing EGFP without the USP10 peptide fused at the C-terminus was constructed at the same time. U2OS cells were transduced with lentiviruses generated with these vectors at a multiplicity of infection (MOI) of 1.0 and stably transduced cells were selected with 1 µg/ml puromycin for 48 h. EGFP and EGFP-USP10(1-40) fusion protein expression was induced in resistant cells for 24 h with 0.5 µg/ml doxycycline, and EGFP-positive cells were sorted at the Dalhousie University Flow Cytometry Core facility. Sorted cells were propagated for 2 passages without doxycycline and re-tested for doxycycline-regulated EGFP and EGFP-USP10(1-40) fusion protein expression.

### Gene silencing and generation of knockout cell lines using CRISPR/Cas9

For GCN2 silencing, U2OS cells were transfected with Ambion Silencer Select Pre-designed siRNAs (s54067, siGCN2-1; or s54069, siGCN2-2) using Lipofectamine RNAiMAX according to manufacturer's protocol (reverse transfection in 12-well cluster dishes) and treated/analysed at 48 h post-transfection. For a non-targeting siRNA control, cells were transfected with Ambion Silencer Select Negative Control #2 (Cat. 4390846).

The lentiCRISPR-v2 plasmid was Addgene plasmid #52961 (deposited by Feng Zhang; RRID:Addgene_52961) (Sanjana et al., 2014). The lentiCRISPR-v2 plasmids encoding guide RNAs targeting human GCN2 and PKR were cloned with primer sequences designed using Broad Institute GPP Web Portal (https://portals.broadinstitute.org/gpp/public/analysis-tools/sgrna-design). Guide RNA insert sequences were: GCN2, 5′-AACTGGCCAAGAAACACTGTGGG-3′; PKR, 5′-GCAACCTACCTCCTATCATGTGG-3′. The lentiCRISPR-v2 plasmids encoding guide RNAs targeting human G3BP1 and G3BP2 genes were a kind gift from Dr Adrianne Weeks (Dalhousie University, Halifax, NS, Canada). Guide RNA insert sequences were: G3BP1 5′-AAGCCTAGTCCCCTGCTGGTCGG-3′; G3BP2 5′-TGGCCATAAACAGCTTCCTGGGG-3′. U2OS cells were transduced with lentiviruses generated with these vectors at an MOI of 1.0, and stably transduced cells were selected with 1 µg/ml puromycin for 48 h. Resistant cells were seeded onto 12-well cluster dishes and used in experiments at 48 h post-seeding (4 days post-transduction with lentiviruses) or seeded onto 96-well dishes for singe-cell clone isolation. Knockout clones were confirmed using western blotting and subsequently used in experiments.

### Immunofluorescence staining

Cell fixation and immunofluorescence staining were performed according to the procedure described in Kedersha and Anderson (2007). Briefly, cells grown on 18-mm round coverslips were fixed with 4% paraformaldehyde in PBS for 15 min at ambient temperature and permeabilized with cold methanol for 10 min. After 1-h blocking with 5% bovine serum albumin (BSA, BioShop, Burlington, ON, Canada) in PBS, staining was performed overnight at +4°C with antibodies to the following targets: 4E-BP (1:1600; rabbit, Cell Signaling, #9644); eIF3B (1:400; rabbit, Bethyl Labs., A301-761A); eIF4G (1:200; rabbit, Cell Signaling, #2498); FMRP (1:200; rabbit, Cell Signaling, #7104); G3BP1 (1:400; mouse, BD Transduction, 611126); G3BP2 (1:1000; rabbit, Millipore Sigma, HPA018304); GCN2 (1:1000; rabbit, Cell Signaling, #65981); HuR (1:200; mouse, Santa Cruz Biotechnology, sc-5261); PABP (1:200; mouse, Santa Cruz Biotechnology, sc-32318); phospho-GCN2 (1:200; rabbit, Cell Signaling, #94668); RPS6 (1:100; mouse, Cell Signaling, #2317); TIA-1 (1:200; goat, Santa Cruz Biotechnology, sc-1751); TIAR (1:1000; rabbit, Cell Signaling, #8509). Alexa Fluor (AF)-conjugated secondary antibodies used were: donkey anti-goat IgG AF488 (Invitrogen, A11055); donkey anti-rabbit IgG AF488 (Invitrogen, A21206); donkey anti-mouse IgG AF555 (Invitrogen, A21202); donkey anti-rabbit IgG AF555 (Invitrogen, A31572); donkey anti-goat IgG AF647 (Invitrogen, A32839); or goat anti-rabbit IgG AF647 (Invitrogen, A21245). Where indicated, nuclei were stained with Hoechst 33342 dye (Invitrogen, H3570). Slides were mounted with ProLong Diamond Antifade Mountant (Invitrogen, P36970) and imaged using Zeiss AxioImager Z2 fluorescence microscope and Zeiss ZEN 2011 software. Quantification of GF-positive cells was performed by counting the number of cells with at least two discrete cytoplasmic foci co-stained with two markers from at least 3 randomly selected fields of view, analysing >100 cells per treatment in each replicate.

### Western blotting

Whole-cell lysates were prepared by direct lysis of PBS-washed cell monolayers with 1× Laemmli sample buffer (50 mM Tris-HCl pH 6.8, 10% glycerol, 2% SDS, 100 mM DTT, 0.005% Bromophenol Blue). To preserve phosphorylation status of proteins, following 5-min agitation at ambient temperature, lysates were immediately placed on ice, homogenized by passing through a 21-gauge needle, and stored at −20°C. Aliquots of lysates thawed on ice were incubated at 95°C for 3 min, cooled on ice, separated using denaturing PAGE, transferred onto PVDF membranes using Trans Blot Turbo Transfer System with RTA Transfer Packs (Bio-Rad Laboratories, Hercules, CA, USA) according to manufacturer's protocol and analysed by immunoblotting using antibody-specific protocols. Antibodies to the following targets were used: 4E-BP (1:1000; rabbit, Cell Signaling, #9644); β-actin (1:2000; HRP-conjugated, mouse, Santa Cruz Biotechnology, sc-47778); eIF2α (1:1000; rabbit, Cell Signaling, #5324); eIF3A (1:1000; rabbit, Cell Signaling, #3411); eIF3B (1:2000; rabbit, Bethyl Labs., A301-761A); eIF4E (1:1000; rabbit, Cell Signaling, #2067); eIF4G (1:1000; rabbit, Cell Signaling, #2498); G3BP1 (1:4000; mouse, BD Transduction, 611126); G3BP2 (1:2500; rabbit, Millipore Sigma, HPA018304); GCN2 (1:1000; rabbit, Cell Signaling, #3302); GFP (1:1000; rabbit, Cell Signaling, #2956); PKR (1:1000; rabbit, Cell Signaling, #3072); phospho-T37/T46-4E-BP (1:1000; rabbit, Cell Signaling, #2855); phospho-S51-eIF2α (1:1000; rabbit, Cell Signaling, #3398); phospho-T899-GCN2 (1:1000; rabbit, Epitomics, 2425-1); phospho-S235/S236-S6 (1:1000; rabbit, Cell Signaling, #2211); ribosomal protein S6 (RPS6; 1:2000; mouse, Cell Signaling, #2317). For band visualization, HRP-conjugated anti-rabbit IgG (Goat, Cell Signaling, #7074) or anti-mouse IgG (Horse, Cell Signaling, #7076) were used with Clarity Western ECL Substrate on the ChemiDoc Touch Imaging Sysytem (Bio-Rad Laboratories, Hercules, CA, USA).

### Ribopuromycylation assay

The puromycin incorporation assay was performed as described in Panas et al. (2015b) with the following modifications. Puromycin was added to the medium at the final concentration of 10 µg/ml for 10 min. Cells were washed with PBS and the whole-cell lysate preparation and western blotting analysis were done as described above. For electrophoresis, samples were loaded onto Mini-PROTEAN TGX Pre-cast Stain-Free gels (5-15%, Bio-Rad Laboratories, Hercules, CA, USA) and total protein was visualised post-transfer to PVDF membranes on ChemiDoc Touch Imaging System. Puromycin incorporation into nascent polypeptides was visualised using anti-puromycin antibody (1:6000; mouse, MilliporeSigma, MABE343), quantified using Bio-Rad Image Lab 5.2.1 software and values normalised to the StainFree signal for each lane.

### m^7^GTP-agarose pulldown assay

The m^7^GTP cap pulldown assay was performed as described in Fujimura et al., (2012), except we used γ-aminophenyl-m^7^GTP agarose (AC-155, Jena Biosciences GmbH, Jena, Germany). Briefly, cells grown in 10-cm dishes were lysed with buffer containing 50 mM Tris-HCl pH 7.4, 100 mM NaCl, 1 mM EDTA, 0.5% Igepal (NP-40 substitute), and protease/phosphatase inhibitor cocktail (Cell Signaling, #5872). To decrease non-specific binding to beads, lysates were pre-cleared using blank agarose (AC-001, Jena Biosciences GmbH, Jena, Germany) for 15 min at +4°C prior to γ-aminophenyl-m^7^GTP agarose pulldown. Binding was performed for 2 h at +4°C with rotation, followed by three washes with lysis buffer and elution with 1× Laemmli sample buffer for 10 min at +65°C.

### Co-immunoprecipitation using anti-eIF3B antibody

Cells grown in 10-cm dishes were lysed with ice-cold buffer containing 50 mM Tris-HCl pH 7.6, 140 mM NaCl, 1.5 mM MgCl_2_, 0.5% Igepal (NP-40 substitute), and protease/phosphatase inhibitor cocktail (Cell Signaling, #5872). After 5 min, cells were scraped and incubated for 5 min at +4°C with rotation. Lysates were clarified for 5 min at 10,000 ***g*** at +4°C and protein concentration was determined using a DC Protein Assay (Bio-Rad Laboratories, Hercules, CA). For immunoprecipitation, 1 µg of rabbit anti-eIF3B antibody (1:100; Bethyl Labs, A301-761A) was added to each lysate containing 0.5 mg of protein in 750 µl volume and incubated for 1 h at +4°C with rotation. Then, 75 µl of protein G magnetic bead suspension (S1430, NEB) pre-equilibrated in lysis buffer was added to each tube and incubation continued for an additional 1 h at +4°C. Beads were concentrated using a magnetic rack (SureBeads Magnetic Rack, Bio-Rad), supernatant removed, and beads were washed three times with 500 µl of lysis buffer using a magnetic rack according to manufacturer's protocol. Co-immunoprecipitated proteins were eluted from the beads using 150 µl of 1× Laemmli sample buffer for 5 min at +75°C and analysed by western blotting as described above except the HRP-conjugated mouse anti-rabbit native IgG secondary antibody (Cell Signaling, #5127) was used to prevent visualisation of heavy and light chains of co-eluted antibody.

### Live microscopy

A549[EGFP-G3BP1] cells (Khaperskyy et al., 2012) were seeded onto six-well cluster dishes in DMEM without Phenol Red supplemented with 10% fetal bovine serum and 2 mM L-glutamine at 37°C in 5% CO_2_ atmosphere. At 24 h post-seeding, cells were treated as indicated and individual fields of view were imaged using EVOS Cell Imaging System (Thermo Fisher Scientific) at room temperature (<2 min) and promptly returned to 37°C incubator for each time point.

### Statistical analysis

All numerical values are plotted as means calculated from three independent biological replicates (separate experiments performed on different days); the error bars represent standard deviations. Statistical analysis of each data set is described in figure legends and was performed using GraphPad Prism 8 software. Asterisks denote *P* values as follows: *P<0.05; **P<0.01; ***P<0.001.

## Supplementary Material

Supplementary information

Reviewer comments
