# Supplementary material for: UV damage induces G3BP1-dependent stress granule formation that is not driven by mTOR inhibition-mediated translation arrest
Source: J Cell Sci. 2020 Oct 28;133(20):jcs248310. doi: 10.1242/jcs.248310 (PMC7648617; doi:10.1242/jcs.248310)
Supplement: Supplementary information [file joces-133-248310-s1.pdf]

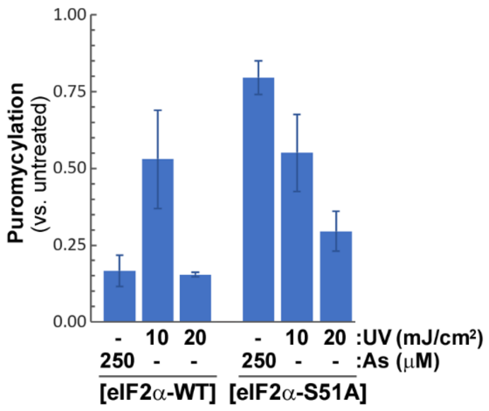

Figure S1. Stress-induced translation arrest in mouse embryonic fibroblasts. Protein synthesis rates were measured in mouse embryonic fibroblasts engineered to express either wild-type or S51A mutant eIF2α ([eIF2α-WT] or [eIF2α-S51A], respectively) that were treated with sodium arsenite (As) or UV light (UV) using ribopuromycylation assay as presented in Figure 2B. Total signal intensity was quantified and normalised to total protein content in each lane as visualised using Stain-Free reagent (N = 3). Error bars represent standard deviation.

Journal of Cell Science • Supplementary information

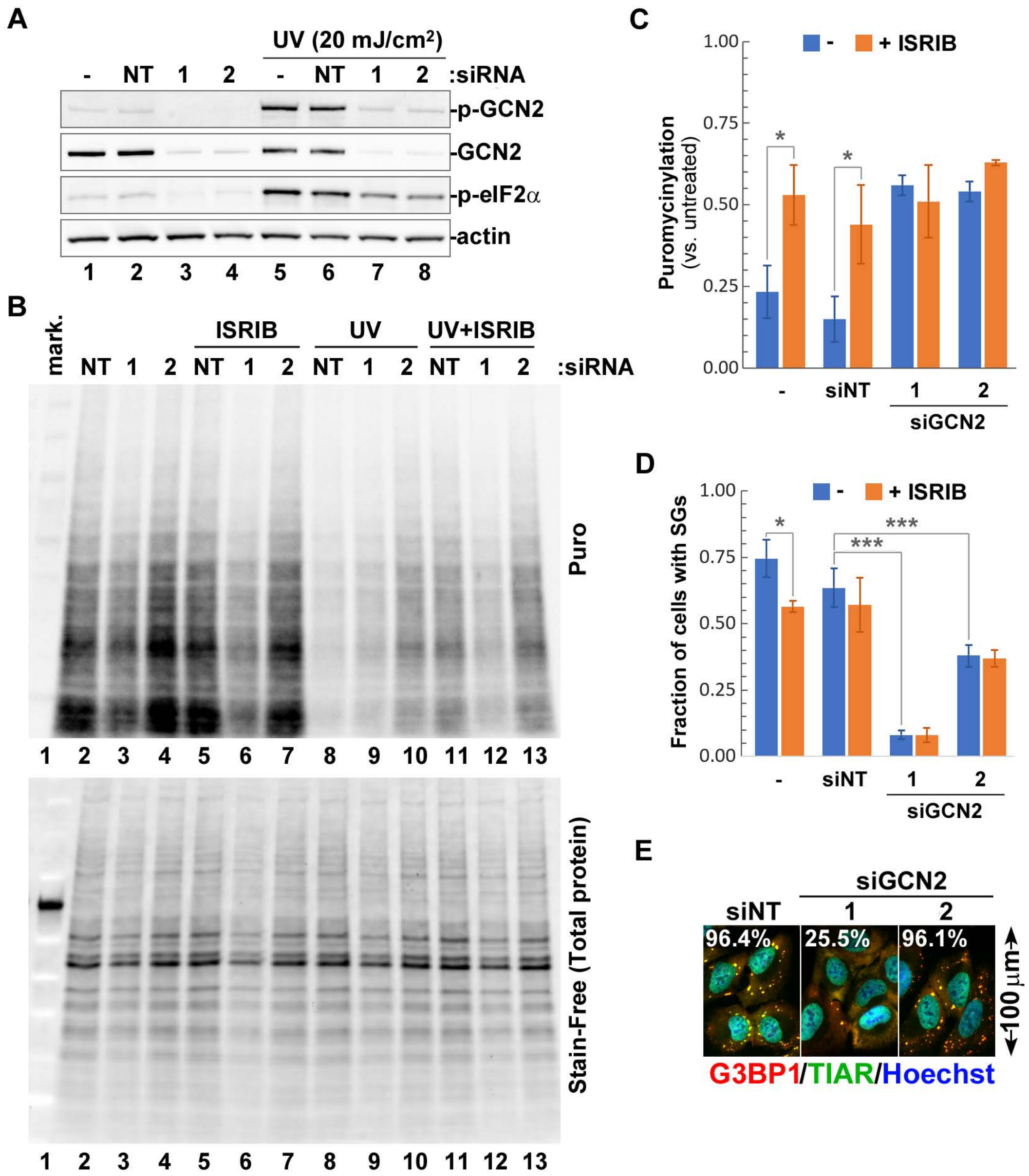

Figure S2. UV-induced SG formation is enhanced by GCN2. U2OS cells were transfected with two different siRNAs targeting GCN2 (1 or 2), non-targeting siRNA (NT), or left untransfected, and were analysed after 48 h for GCN2 expression levels, UV-induced translation arrest and SG formation. (A) Effects of GCN2 silencing on eIF2α phosphorylation in response to UV light (UV) were analysed by western bloting. Efficiency of siRNA-mediated knock down was monitored by staining for total and phosphorylated GCN2. Staining for actin was used as loading control. (B-E) Effects of GCN2 and GCN2-mediated eIF2α phosphorylation on translation arrest and SG formation were analysed in cells incubated with or without ISRIB for 2 h post-UV exposure. (B) Protein synthesis rates were analysed using ribopuromycylation assay and western blotting with anti-puromycin antibody (Puro, top panel). Total protein content in each lane was visualised using Stain-Free reagent (bottom panel). (C) UV-induced translation arrest was quantified from ribopuromycylation assays (N = 3). Error bars represent standard deviation; *$p < 0.05$ (Two-way ANOVA followed by Tukey's multiple comparisons test). (D) UV-induced SG formation was quantified from immunofluorescence microscopy staining (N = 3). Error bars represent standard deviation; *$p < 0.05$; **$p < 0.01$; ***$p < 0.001$ (Two-way ANOVA followed by Tukey's multiple comparisons test). (E) SG formation in cells treated with sodium arsenite was analysed by staining for G3BP1 (red) and TIAR (green). Nuclei were stained with Hoechst dye (blue). Fraction of cells with SGs was quantified and is indicated on the panels (average value, N=2).

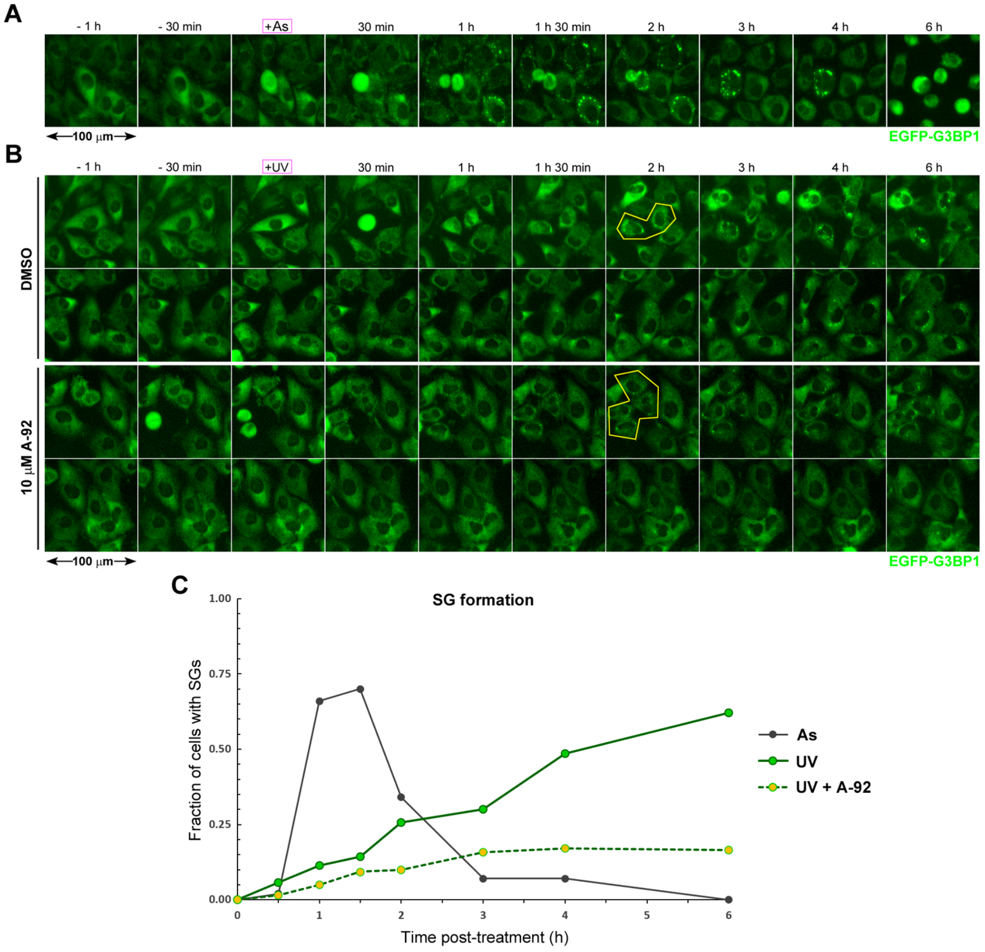

Figure S3. Time course of SG formation in live A549 cells stably expressing EGFP-G3BP1 reporter. (A,B) A549[EGFP-G3BP1] cells were imaged at indicated times before and after treatment with (A) 0.5 mM sodium arsenite or (B) 20 mJ/cm^2 UV light in media containing 5 μM A-92 GCN2 inhibitor of DMSO vehicle control. Images taken immediately following arsenite addition or UV exposure are labelled "+As" and "+UV", respectively. In panel B, post-mitotic cells are outlined in yellow. (C) Mean fraction of SG-positive cells was quantified from 2 separate biological replicates. At least 100 individual cells were analysed over the time course of the experiment in each treatment.

Journal of Cell Science • Supplementary information

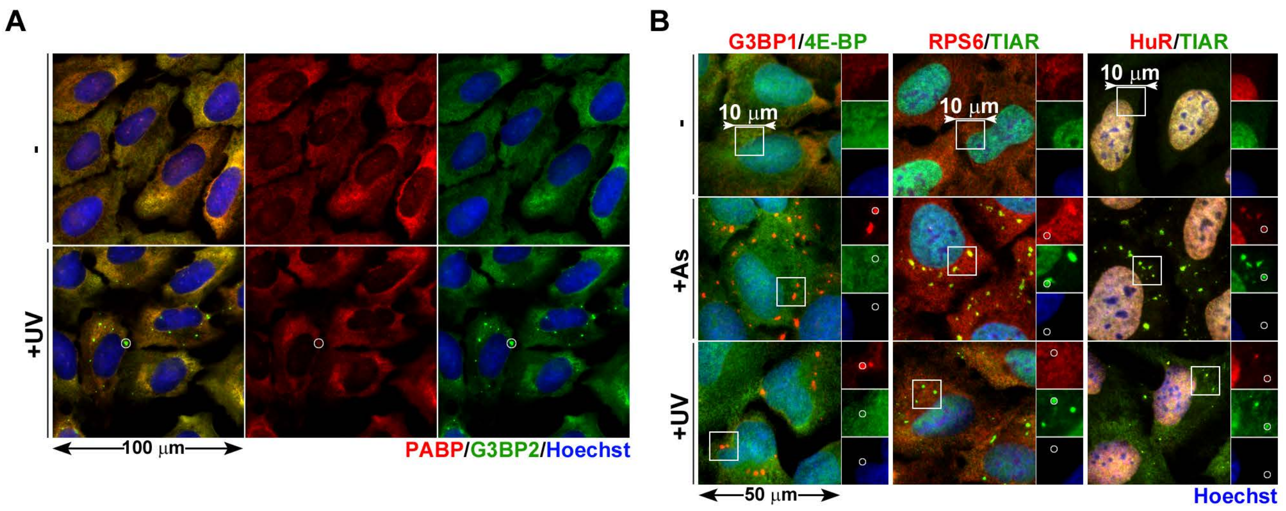

Figure S4. Recruitment of markers to UV-induced SGs. (A) U2OS cells exposed to 20 mJ/cm^2 UV light were fixed at 2 h post-treatment and stained for PABP (red) and G3BP2 (green). Nuclei were stained with Hoechst dye (blue). Representative SG foci are outlined with circles. (B) U2OS cells exposed to 20 mJ/cm^2 UV light (UV) or treated with 0.5 mM sodium arsenite (As) were analysed by immunofluorescence microscopy using staining with antibodies specific for the indicated cellular proteins. Nuclei were stained with Hoechst dye (blue). Representative SG foci in the enlarged outsets are outlined with circles.
