# Supplementary material for: UV damage induces G3BP1-dependent stress granule formation that is not driven by mTOR inhibition-mediated translation arrest
Source: J Cell Sci. 2020 Oct 28;133(20):jcs248310. doi: 10.1242/jcs.248310 (PMC7648617; doi:10.1242/jcs.248310)
Supplement: Reviewer comments [file joces-133-248310_review_history.pdf]

Journal of Cell Science

UV damage induces G3BP1-dependent stress granule formation that is not driven by mTOR inhibition-mediated translation arrest

Shan Ying and Denys A. Khaperskyy
DOI: 10.1242/jcs.248310

Editor: Maria Carmo-Fonseca

Review timeline
Original submission: 29 April 2020
Editorial decision: 2 June 2020
First revision received: 10 August 2020
Editorial decision: 2 September 2020
Second revision received: 9 September 2020
Accepted: 17 September 2020

Original submission

<u>First decision letter</u>

MS ID#: JOCES/2020/248310

MS TITLE: UV damage induces G3BP1-dependent stress granule formation that is not driven by translation arrest via mTOR inhibition

AUTHORS: Shan Ying and Denys Khaperskyy
ARTICLE TYPE: Research Article

We have now reached a decision on the above manuscript.

To see the reviewers' reports and a copy of this decision letter, please go to: https://submit-jcs.biologists.org and click on the 'Manuscripts with Decisions' queue in the Author Area. (Corresponding author only has access to reviews.)

I have now received comments on your manuscript from two experts. As you will see, both thought that the work was potentially quite interesting and significant but all also raised a number of concerns that must be dealt with before the manuscript can be reconsidered. Please address these issues as thoroughly as possible. In particular, you should provide the experimental controls and quantifications indicated by the reviewers. If you think that you can deal satisfactorily with the criticisms on revision, I would be pleased to see a revised manuscript. We would then return it to the reviewers.

We are aware that you may currently be unable to access the lab to undertake experimental revisions. If it would be helpful, we encourage you to contact us to discuss your revision in greater detail. Please send us a point-by-point response indicating where you are able to address concerns raised (either experimentally or by changes to the text) and where you will not be able to do so within the normal timeframe of a revision. We will then provide further guidance. Please also note that we are happy to extend revision timeframes as necessary.

Please ensure that you clearly highlight all changes made in the revised manuscript. Please avoid using 'Tracked changes' in Word files as these are lost in PDF conversion.

© 2020. Published by The Company of Biologists under the terms of the Creative Commons Attribution License (https://creativecommons.org/licenses/by/4.0/).

I should be grateful if you would also provide a point-by-point response detailing how you have dealt with the points raised by the reviewers in the 'Response to Reviewers' box. Please attend to all of the reviewers' comments. If you do not agree with any of their criticisms or suggestions please explain clearly why this is so.

Reviewer 1

Advance summary and potential significance to field

UV damage is one of many ways to induce stress granule (SG) formation although it is relatively understudied compared to other stressors such as sodium arsenite or heat. In this article, Ying and Khaperskyy dissect the mechanism by which UV-induced SGs are nucleated compared to SGs induced by other stimuli. Collectively, the work suggests a new pathway based on phosphorylation of the GCN2 kinase, but independent of eIF2alpha. (This reviewer notes that UV-induced translational inhibition being marginally dependent on eIf2α phosphorylation has been previously reported by Aulas et al, J. Cell Science, 2017, PMID 28096475). Ying et al also show that UV-induced SGs are able to form when either the mTOR or the eIF2alpha pathway is non-functional. Additionally, they describe that UV-induced SGs rely on G3BP1, but not G3BP2, for their formation and require an interaction between G3BP1 and Caprin1 (deduced from its regulation by USP10 N-terminal fragment). Lastly, they show that 48S pre-initiation translation complexes are intact during UV-stress, but they are not themselves components of SGs. This last point is again consistent with the work of Aulas et al (PMID 28096475).

Overall, the paper is well written and the figures are well laid-out. The authors have used U2OS cells as their model, which is broadly popular in the stress granule field. The two aspects that are novel in this paper are the dependence of the UV-induced SG pathway on GCN2, and that the pathway relies on G3BP1, but not G3BP2.

Comments for the author

Essential comments to address for resubmission:
1. Throughout the paper, there are western blots showing P-eIF2alpha and P-GCN2, but blots to indicate total eIF2alpha and GCN2 levels are absent. (Phosphoproteins should be normalized against the total level of that protein). This is essential in order to provide needed quantifications and solidify conclusions that the differences in phosphorylation are significant. (Also true for S6K and 4E-BP).
2. Have the authors quantified whether the UV treatment used impact cell viability? (or can they provide a citation to this point).
3. Figure 1D (p-eIF2alpha, p-S6 blots) appears to be over-contrasted.
4. Presentation of data related to Figure 3 and the two siRNAs for GCN2 could be improved. As written, it feels like a “bait and switch”, with quite some text devoted to siGCN2-1 – only to conclude that much of the effects observed were due to off-target effects.
5. In general, the descriptive immunofluorescence data should be improved by quantification of the stress granules in all figures? (it is done only for some figures). For example, in Figure 4B, it is mentioned that GCN2 inhibition “caused formation of slightly smaller SGs in fewer cells”. This result would be strengthened by quantification. Also, Figures 5A and 5D make important points which would be better supported by quantification.
6. Figure 5: Have the authors used a negative control in the experiment using CRISPR to KO G3BP1 and G3BP2.
7. Discussion is long and could be more focused. As written, there is quite some repetition with the results text.

Comments that would extend the story:
1. Does G3BP2 localize to SGs in UV-induced conditions even though it is not required for nucleation? Also, whether these UV-induced SGs contain other canonical SG markers like Caprin1 or HuR would have been an addition to the collective knowledge pool.
2. What is happening to MRS (other GCN2 target)? Does it contribute to SG formation?

© 2020. Published by The Company of Biologists under the terms of the Creative Commons Attribution License (https://creativecommons.org/licenses/by/4.0/).

3. Can the authors expand on what is the evidence that the mechanism is GCN2 specific? What about PKR and PERK (other kinases that phosphorylate eIF2alpha)? (This last point could be considered in the re-written discussion perhaps….)

Reviewer 2

Advance summary and potential significance to field

In this work, the authors present mostly convincing evidence (protein synthesis assays, microscopy, cap-pull downs +/- presence of TOR inhibiting treatments) that UV-induced SGs do not depend on mTOR inhibition nor disassembly of eIF4F initiation complexes. Furthermore, they show that GCN2 activity, based on siRNA and inhibitor studies, contributes to UV SG induction and translation repression in a manner mostly independent of eIF2a phopsphorylation (though this was partly addressed in prior work by the Ivanov/Anderson lab). Finally UV SG assembly depends on G3BP1, a commonly implicated SG assembly protein, and the mechanism depends on G3BP1's NTF-2 domain, based on co-over-expression of a domain of USP10 that interacts with G3BP1's NTF-2 domain and impairs other interactions of G3BP1 that drive SG assembly.
The quality of work performed in this manuscript is mostly high, well controlled and fair in interpretation. An issue that dampens my enthusiasm somewhat is that many of the findings presented are either negative (i.e. ruling out TOR, eIF2a phopshoryaltion contributions to UV SG induction and translation repression), not that surprising (that G3BP1 is involved in SG assembly, via it's primarily understood means for doing so; NTF2 domain function), or in some cases repetitive with prior work from the Ivanov and Anderson lab. That said, there is interesting data presented regarding eIF4F protein interactions under UV stress, and the indication of a novel Gcn2 function, and it certainly rounds out the topic of UV SG induction a bit more fully than previous work has. I suggest a few possible experimental directions below that would make me more enthusiastic and I look forward to seeing a revision.

Comments for the author

Major points
1.) Some data is repetitive with prior work from Aulas et al, Ivanov lab, JCS 2017. Note, although the authors use a different cell line background conceptually the following datasets/experiments in this work closely mirror work done in that previous paper:
Fig 2A-B translation repression findings in WT and eIF2a S51A mutant cells (same assay, different cells) were also in Ivanov lab paper from 2017 (Figure 3A), albeit Puromycylation assays here are easier to visualize.
Figure 3 B/C: Although not quite the same (GCN2 siRNA KD here v KO cell lines), the basic question of does GCN2 aid translation repression under UV stress was also examined in Aulas et al , with similar ultimate conclusions – Figure 5A-B in the Aulas paper Figure 6: While microscopy images are certainly clearer, 6A and B is experimentally a repeat of work from Aulas et al – Fig 2C

Despite this, the authors clearly cite the prior study and in many instances the effects they describe are better shown so I don't recommend removal of the data. I just raise this point as a general concern on the current novelty of the manuscript.

2.) The authors convincingly show that GCN2 activity underpins SG assembly under UV stress, and as they say on line 342, suggests the involvement of other yet to be identified GCN2 targets. It would really help the paper if they could identify some putative targets as this could be a significant advance into a novel means of GCN2 controlling SG assembly and/or translation. It sounds from their discussion as if nothing is really known about GCN2 phophotargets beyond eIF2a and the tRNA synthetase MRS. This latter target could thus presumably be examined to see if it exerts effects on SG assembly also (I understand their arguments as to why this is likely not ternary complex related, but maybe it reflects some other function??).

Alternatively, if other known targets or GCN2 phosphoproteomic datasets don't exist, the authors could also generate their own GCN2-dependent phosphoproteomic dataset, +/- their GCN2 inhibitor (or try a GCN2 substrate pull down in presence of crosslinkers -

© 2020. Published by The Company of Biologists under the terms of the Creative Commons Attribution License (https://creativecommons.org/licenses/by/4.0/).

https://pubmed.ncbi.nlm.nih.gov/30636329/); this would surely be a useful resource to the signaling/GCN2 community. Note, I clearly realize that either of these suggestions is a substantial body of work potentially (especially at this time), thus I don't insist on the authors doing this; merely a suggestion for future work. Also characterizing any hits obtained in their own, or work of others in any detail wouldn't be critical if they did go down this route.

More doable but still informative: Is the Gcn2 kinase in SGs under UV stress?
Does its kinase activity affect SG assembly rates, disassembly rates, or both (could do stress timecourse experiments with the inhibitor). Basically, any further foray in the direction of what GCN2 is doing would be an improvement.

3.) Another focus for giving the paper more novel data would be to test the importance of SG assembly using their clever Dox-inducible USP10 system to cell survival under UV stress. Alternatively, they could examine other UV or SG-relevant phenotypes such as proper regulation of UV-responsive mRNAs, signaling events, DNA damage assays etc under UV stress, in the presence or absence of SG assembly.

Note, since mRNAs may not be a major part of UV SGs, based on Ivanov's poly(A) staining data and the authors own 4F complex data, the survival test is probably the simpler assay. However, I might suggest the authors check for the presence of de(A) RNA in UV SGs using RNA dyes like SYTO select, just incase de(A) mRNAs are actually what is being regulated (might explain why 4G is absent). The fact that UV SGs do show sensitivity to CHX (Aulas et al, Fig 4A) suggest to me at least that some mRNAs are cycling through UV SGs.

Regarding points 2 and 3 above, my suggestions for minimal revisions/novel additions would be to try the survival and RNA staining experiments in point 3 above (compare the latter to Arsenite SGs?). One of the "GCN2 in SGs / Effects of GCN2 inhibition on SH assembly v disassembly timecourse experiments" (point 2) would be sufficient also. However, going deeper experimentally as I've suggested, or other things along these lines would certainly be welcome if the authors are motivated/able to try under current circumstances.

Minor points

Certain microscopy and western blots not currently quantitated should be (or in one case, raw data not shown); some discussed in more detail below but to summarize:
Fig 1C (Microscopy); Fig 1D/A (either or; primarily to assess non-P and p-4E-BP levels, though eIF2a, S6K etc also could be quantified); Fig 2B; gels shown but not quantification; Fig 2C (quantification shown but not actual gels); Fig 2D (Microscopy), Fig 3F (Microscopy), Fig 4D (Microscopy); Fig 5A (Microscopy)
- any/all of these could suffice as supplementary material.

line 48: I would soften/alter statement to reflect idea that some type of non-standard 48S pre-initiation complex may exist in SGs. As the authors likely know, the notion that SGs harbor 48S pre-initiation complexes is not based on any actual knowledge of the mRNP state for any given mRNA, simply the fact that various initiation factor proteins are present in SGs. Additionally, the ref cited (Kedersha et al, 2002, MBC) makes the point that arsenite SGs lack eIF2 hence if 48S complexes of a sort are present, they are already non-canonical by absence of this complex.

Fig1 A, line 235: I indeed see a 3rd higher molecular weight Phospho band for 4E-BP following UV as authors state; however overall level of phosphorylation appears lower, at least in 1A. This could mean more 4E-BP may now be de-phosphorylated and thus competent for translation repression (meaning TOR would potentially play a role in UV stress translation repression); alternatively 4E-BP levels could simply be reduced? The authors should assess this with a regular 4E-BP Ab western.

Note, in Fig 1D (lanes 1 and 2), it actually looks to me as though total 4EBP levels increase under UV stress (cumulative band intensity for 4EBP in UV v control v actin as normalization). Perhaps the authors could quantitate from this gel (normalized to actin), or simply conditions in 1A with 4E-BP staining.
Either way, I think it's important to quantify here.

© 2020. Published by The Company of Biologists under the terms of the Creative Commons Attribution License (https://creativecommons.org/licenses/by/4.0/).

Line 247, Fig 1C: Minor point, but why did authors suspect TOR inhibition might “prevent” UV SG assembly; couldn’t it equally have enhanced? Does it? (i.e. are there any quantitative differences in SG number, size etc for SG foci shown in 1C?). More generally, authors may want to clearly state their experiment also assesses whether TOR inhibition per se, often heralded as a master regulator of translation, is capable of inducing SGs; obviously the answer is no.

Line 259: Minor point, can ignore but I found wording here confusing. I would just edit to be more explicit and say that 4G no longer binds/pulls down with m7GTP cap, reflecting impairment of 4E-4G interaction. “..dissociation of 4E and 4G..” confused me because obviously 4E doesn’t dissociate from the m7GTP cap, which is the basis of the assay, which isn’t looking at eIF4G-eIF4E interaction directly.

Line 276, Fig 2B: Based on authors own data (and later more accurate statement), I’d soften conclusion on line 276 to “largely” phospho-eIF2a independent.

Line 89/Figure 2D: It would strengthen author’s conclusion (e.g. “just as well”) if actual quantitation of SG foci was presented rather than just images.
This could be cells with foci (as in 3E), average number of cells with foci intensity, size etc; I leave it up to the authors to choose the most practical informative measure.

Figure 3 / siRNA 1 for GCN2: While very honest, I wonder if it serves the authors to show data where two KDs for GCN2 have different effects (3B, E and F), especially since siRNA 1 already exhibits slow growth and protein synthesis, before the addition of stress etc. I would suggest they just show siRNA KD 2, unless they are able to determine anything mechanistically about why their siRNA1 data is distinct.

I would consider quantifying Figure 4D. Also, does G3BP1 show any particular perinuclear re-localization in UV stress with USP10 peptide expression; sort of looks like this in images shown, but may just be unrepresentative.

Figure 5A: It does appear that cells lacking G3BP1 are impaired in UV SG assembly, relative at least to Se and Arsenite treated cells; however, fewer UV SGs form relative to Arsenite stress in the first place (e.g. see Fig 6A).
Thus, it would certainly help if the authors compare to a WT cell control subject to UV stress to make clear to what extent G3BP1-deficient cells are impaired in SG assembly. Some arbitrary threshold could be used to focus on quantifying the cells most depleted in G3BP1 for this purpose. Additionally, because this experimental KO approach likely yields a mixed population of cells that still express G3BP1 or 2, it makes interpreting the G3BP2 KO data more difficult if the authors can’t actually visualize G3BP2.
They state for example the Se stress results in no SGs forming in G3BP2 KO cells; does this mean the presumed small population of WT cells in this culture are deficient in Se-SG induction due to neighboring cell effects? Or is the G3BP2 KO actually much more efficient? If the authors can’t discriminate, I’d either acknowledge these issues, or simply not show the G3BP2 KO microscopy data.

First revision

<u>Author response to reviewers' comments</u>

Reviewer 1
Advance Summary and Potential Significance to Field...
UV damage is one of many ways to induce stress granule (SG) formation although it is relatively understudied compared to other stressors such as sodium arsenite or heat. In this article, Ying and Khaperskyy dissect the mechanism by which UV-induced SGs are nucleated compared to SGs induced by other stimuli. Collectively, the work suggests a new pathway based on phosphorylation of the GCN2 kinase, but independent of eIF2alpha. (This reviewer notes that UV-induced

© 2020. Published by The Company of Biologists under the terms of the Creative Commons Attribution License (https://creativecommons.org/licenses/by/4.0/).

translational inhibition being marginally dependent on eIf2α phosphorylation has been previously reported by Aulas et al, J. Cell Science, 2017, PMID 28096475). Ying et al also show that UV-induced SGs are able to form when either the mTOR or the eIF2alpha pathway is non-functional. Additionally, they describe that UV-induced SGs rely on G3BP1, but not G3BP2, for their formation and require an interaction between G3BP1 and Caprin1 (deduced from its regulation by USP10 N-terminal fragment). Lastly, they show that 48S pre- initiation translation complexes are intact during UV-stress, but they are not themselves components of SGs. This last point is again consistent with the work of Aulas et al (PMID 28096475).

Overall, the paper is well written and the figures are well laid-out. The authors have used U2OS cells as their model, which is broadly popular in the stress granule field. The two aspects that are novel in this paper are the dependence of the UV-induced SG pathway on GCN2, and that the pathway relies on G3BP1, but not G3BP2.

We thank Reviewer 1 for their assessment of our work that aimed at understanding the formation of SGs following UV stress. Indeed, several observations made previously by others, including an important study by Aulas et al. (2017), are also featured in our manuscript. Most obviously, we confirm that UV-induced SG formation does not rely on eIF2alpha phosphorylation, and that some of the components of preinitiation complexes (eIF3B and eIF4G) are not accumulating in UV-induced SGs. These observations are not presented in our work as novel, but we believe that these experiments are important because they (a) verify and confirm previous studies (which is an important function of scientific inquiry); (b) validate our experimental system and approach; (c) provide necessary context for understanding the contribution of GCN2 catalytic activity, which is most often viewed as directed exclusively towards eIF2alpha phosphorylation. In fact, the very goal of our work was to gain more understanding about UV-induced SG formation that based on these previous studies appeared distinct from SG formation triggered by other types of stress. Accordingly, the study by Aulas et al. and other relevant studies are cited numerous times throughout the text. As Reviewer 1 notes, the focus of our work is on the contribution of mTOR signaling, GCN2 activation, and the role of SG-nucleating proteins in UV-induced SG formation, which to our knowledge were not systematically analysed previously.

We appreciate valuable comments and advice from Reviewer 1 on the ways to strengthen our manuscript. We believe that the revised version that incorporates majority of suggestions by reviewers 1 and 2 represents considerable improvement compared to the original submission. In revised manuscript, we present results of additional experiments that further support the main conclusions of our study. Inclusion of new data resulted in addition of Supplementary figures and significant rearrangements of figures 3, 4, and 5, with some panels from original figures now moved to supplementary figures. In addition to text modifications and changes to the presentation of original data (e.g. inclusion of WB controls and SG quantitation), in this revised manuscript we present:

1) Analysis of stable clonally selected GCN2, PKR, G3BP1, and G3BP2 CRISPR/Cas9 knockout cell lines (presented in new figures 4 and 5).
2) Analysis of SG formation at additional later time point (6 h) in the GCN2 deficient and the parental U2OS cell lines (new figure 4) and in live A549[EGFP-G3BP1] reporter cells over the course of 6 h (new supplementary figure S2) that provide more insights into the role of GCN2 in UV-induced SG formation in interphase cells.
3) Immunofluorescence microscopy staining examining recruitment of G3BP2, PABP, 4E-BP, RPS6, HuR, GCN2 and phospho-GCN2 to UV-induced SGs.

Our responses to specific comments are presented below.

Reviewer 1
Comments for the Author...

Essential comments to address for resubmission:
1. Throughout the paper, there are western blots showing P-eIF2alpha and P-GCN2, but blots to indicate total eIF2alpha and GCN2 levels are absent. (Phosphoproteins should be normalized against the total level of that protein). This is essential in order to provide needed quantifications and solidify conclusions that the differences in phosphorylation are significant. (Also true for S6K and 4E-BP).

© 2020. Published by The Company of Biologists under the terms of the Creative Commons Attribution License (https://creativecommons.org/licenses/by/4.0/).

Although in most cases the differences in phospho-specific band intensities were sufficiently robust to indicate sharp changes in P-eIF2alpha, P-GCN2, P-S6K, and P-4E-BP that were very consistent with expected treatment outcomes, we agree that it is important to include total protein controls in case they change in the course of experiments. We included total protein blots for GCN2, eIF2alpha, S6, 4E-BP, and additional control (arsenite treatment) in the new panel 1A, and total eIF2a in the new panel 1E and all new western blots in the revised manuscript. We did not aim at quantifying phosphorylation of eIF2alpha or other phosphoproteins because these blots served as qualitative indicators of phosphorylation status and we did not attempt to corelate signal strength to the magnitudes of any of the measured outcomes.

2. Have the authors quantified whether the UV treatment used impact cell viability? (or can they provide a citation to this point).
It is well established that UVC exposure at the doses used in our analyses result in cell cycle arrest at early times post-exposure (1-6 h) and cell death at later times post-exposure (24-48 h). At later times (48 h) doses as low as 3 mJ/cm2 can result in 90% cell death (Moutaoufik et al, 2014), and in our observations following 20 mJ/cm2 treatment up to 40% of cells round up and detach from the dish at 24 h post-exposure. Aulas et al. (2017) observed only 20% cell death at 24 h post-exposure in HAP1 cells using the same dose. In the present study, similar to other studies analysing SG formation, we focused on early events and our new analyses at 1-6 h post- UV exposure clearly indicates (Fig. 4A and Fig. S2) that both U2OS and A549 cells remain viable and do not change morphology over this time course. By contrast, 500 uM sodium arsenite treatment (the most widely used way to induce SGs) caused 90% cell rounding and death at 6 h (Fig. S2). Previous studies analysed the impact of eIF2alpha phosphorylation on UV-induced cell death. Now that we discovered new tolls for modulating SG formation post-UV exposure (e.g. USP10(1-40) overexpression) we intend to directly assess contribution of SGs to cell cycle arrest and survival in a follow up study.

3. Figure 1D (p-eIF2alpha, p-S6 blots) appears to be over-contrasted.
High contrast in some of the western blot panels was a result of automatic exposure adjustment in Bio-Rad ImageLab software prior to export to TIFF files. We have now reduced the contrast on these panels (new panel 1E) and all similarly contrasted panels throughout the manuscript from raw ChemiDoc .scn files as per journal’s requirement.

4. Presentation of data related to Figure 3 and the two siRNAs for GCN2 could be improved. As written, it feels like a “bait and switch”, with quite some text devoted to siGCN2-1 – only to conclude that much of the effects observed were due to off-target effects.
We agree that our attempt to be thorough in the description of commercially available siRNA constructs could be perceived as misleading. Accordingly, we removed results for siRNA-1 from the Figure 3 and added new figure (Figure 4) describing experiments using complete GCN2 knock out line that we generated using CRISPR/Cas9 technology. However, we feel that it is important to report the off-target effects of the GCN2 siRNA-1 to the field and retained the original Figure 3 as a supplementary figure (Fig. S1).

5. In general, the descriptive immunofluorescence data should be improved by quantification of the stress granules in all figures? (it is done only for some figures). For example, in Figure 4B, it is mentioned that GCN2 inhibition “caused formation of slightly smaller SGs in fewer cells”. This result would be strengthened by quantification. Also, Figures 5A and 5D make important points which would be better supported by quantification.
We agree and now include quantifications of SGs in revised and new figures.

6. Figure 5: Have the authors used a negative control in the experiment using CRISPR to KO G3BP1 and G3BP2.
We now include new analysis of stable CRISPR KO lines lacking G3BP1, G3BP2, or PKR as control (Fig. 5).

7. Discussion is long and could be more focused. As written, there is quite some repetition with the results text.
We have trimmed our discussion somewhat to reduce repetition from the results text.

© 2020. Published by The Company of Biologists under the terms of the Creative Commons Attribution License (https://creativecommons.org/licenses/by/4.0/).

Comments that would extend the story:
1. Does G3BP2 localize to SGs in UV-induced conditions even though it is not required for nucleation? Also, whether these UV-induced SGs contain other canonical SG markers like Caprin1 or HuR would have been an addition to the collective knowledge pool.

We have included analysis of G3BP2 and HuR localization to UV-induced SGs, as well as 4E-BP, S6, and total and phospho-GCN2, in Figures S3 and Fig. 4D,E.

2. What is happening to MRS (other GCN2 target)? Does it contribute to SG formation?

This is a very good question. However, we believe it is beyond the scope of this work. We plan to examine contribution of MRS to UV-induced SG formation and screen for other potential GCN2 targets in a follow up study.

3. Can the authors expand on what is the evidence that the mechanism is GCN2 specific? What about PKR and PERK (other kinases that phosphorylate eIF2alpha)? (This last point could be considered in the re-written discussion perhaps….)

It is previously well established that PKR and PERK are not involved in UV-induced eIF2alpha phosphorylation (Deng et al., 2002). However, we now include PKR knock out in our analyses, that clearly shows no significant effect of this kinase on SG formation (Fig. 5).

Reviewer 2
Advance Summary and Potential Significance to Field...

In this work, the authors present mostly convincing evidence (protein synthesis assays, microscopy, cap-pull downs +/- presence of TOR inhibiting treatments) that UV-induced SGs do not depend on mTOR inhibition nor disassembly of eIF4F initiation complexes. Furthermore, they show that GCN2 activity, based on siRNA and inhibitor studies, contributes to UV SG induction and translation repression in a manner mostly independent of eIF2a phopsphorylation (though this was partly addressed in prior work by the Ivanov/Anderson lab). Finally, UV SG assembly depends on G3BP1, a commonly implicated SG assembly protein, and the mechanism depends on G3BP1's NTF-2 domain, based on co-over-expression of a domain of USP10 that interacts with G3BP1's NTF-2 domain and impairs other interactions of G3BP1 that drive SG assembly.
The quality of work performed in this manuscript is mostly high, well controlled and fair in interpretation. An issue that dampens my enthusiasm somewhat is that many of the findings presented are either negative (i.e. ruling out TOR, eIF2a phopshoryaltion contributions to UV SG induction and translation repression), not that surprising (that G3BP1 is involved in SG assembly, via it's primarily understood means for doing so; NTF2 domain function), or in some cases repetitive with prior work from the Ivanov and Anderson lab. That said, there is interesting data presented regarding eIF4F protein interactions under UV stress, and the indication of a novel Gcn2 function, and it certainly rounds out the topic of UV SG induction a bit more fully than previous work has. I suggest a few possible experimental directions below that would make me more enthusiastic and I look forward to seeing a revision.

We thank Reviewer 2 for their assessment of our work that aimed at understanding the formation of SGs following UV stress. Indeed, several observations made previously by others, including an important study by Aulas et al. (2017), are also featured in our manuscript. Most obviously, we confirm that UV-induced SG formation does not rely on eIF2alpha phosphorylation, and that some of the components of preinitiation complexes (eIF3B and eIF4G) are not accumulating in UV-induced SGs.
We are glad that Reviewer 2 recognises that the quality of the work presented in our manuscript is high and that it characterises SG induction by UV more fully than previous studies. Indeed, our analyses of mTOR pathway contribution to SG formation yielded negative results, however we disagree that this in any way decreases the value of these findings. The focus of our work is on the contribution of mTOR signaling, GCN2 activation, and the role of SG- nucleating proteins in UV-induced SG formation, which to our knowledge were not systematically analysed previously.
We appreciate valuable comments and advice from Reviewer 2 on the ways to strengthen our manuscript. We believe that the revised version that incorporates majority of suggestions by reviewers 1 and 2 represents considerable improvement compared to the original submission. In

© 2020. Published by The Company of Biologists under the terms of the Creative Commons Attribution License (https://creativecommons.org/licenses/by/4.0/).

revised manuscript, we present results of additional experiments that further support the main conclusions of our study. Inclusion of new data resulted in addition of Supplementary figures and significant rearrangements of figures 3, 4, and 5, with some panels from original figures now moved to supplementary figures. In addition to text modifications and changes to the presentation of original data (e.g. inclusion of WB controls and SG quantitation), in this revised manuscript we present:

1) Analysis of stable clonally selected GCN2, PKR, G3BP1, and G3BP2 CRISPR/Cas9 knockout cell lines (presented in new figures 4 and 5).

2) Analysis of SG formation at additional later time point (6 h) in the GCN2 deficient and the parental U2OS cell lines (new figure 4) and in live A549[EGFP-G3BP1] reporter cells over the course of 6 h (new supplementary figure S2) that provide more insights into the role of GCN2 in UV-induced SG formation in interphase cells.

3) Immunofluorescence microscopy staining examining recruitment of G3BP2, PABP, 4E-BP, RPS6, HuR, GCN2 and phospho-GCN2 to UV-induced SGs.

Our responses to specific comments are presented below.

Reviewer 2
Comments for the Author...
Major points
1.) Some data is repetitive with prior work from Aulas et al, Ivanov lab, JCS 2017. Note, although the authors use a different cell line background, conceptually the following datasets/experiments in this work closely mirror work done in that previous paper:

Fig 2A-B translation repression findings in WT and eIF2a S51A mutant cells (same assay, different cells) were also in Ivanov lab paper from 2017 (Figure 3A), albeit Puromycylation assays here are easier to visualize.
Figure 3 B/C: Although not quite the same (GCN2 siRNA KD here v KO cell lines), the basic question of does GCN2 aid translation repression under UV stress was also examined in Aulas et al , with similar ultimate conclusions – Figure 5A-B in the Aulas paper
Figure 6: While microscopy images are certainly clearer, 6A and B is experimentally a repeat of work from Aulas et al – Fig 2C.

Despite this, the authors clearly cite the prior study and in many instances the effects they describe are better shown so I don't recommend removal of the data. I just raise this point as a general concern on the current novelty of the manuscript.

Indeed, in our study we confirm previous reports that UV-induced SG formation is phospho-eIF2alpha independent, that the GCN2 activity is important for UV-induced translation inhibition, and that some of the components of preinitiation complexes (eIF3B and eIF4G) are not accumulating in UV-induced SGs. Accordingly, these observations are not presented in our work as novel, but we believe that the inclusion of these experiments is important because they (a) verify and confirm previous studies on this topic (which is an important function of scientific inquiry); (b) validate our experimental system and approach as they compare to the published studies on this topic; (c) provide necessary context for understanding the contribution of GCN2 catalytic activity, which is most often viewed as directed exclusively towards eIF2alpha phosphorylation. In fact, the very goal of our work was to gain more understanding about UV-induced SG formation that based on previous studies appeared distinct from SG formation triggered by other types of stress. Furthermore, we complement these previous studies with careful assessment of the magnitude of translation arrest and its correlation with SG formation. To our knowledge, this have not been done previously.

2.) The authors convincingly show that GCN2 activity underpins SG assembly under UV stress, and as they say on line 342, suggests the involvement of other yet to be identified GCN2 targets. It would really help the paper if they could identify some putative targets as this could be a significant advance into a novel means of GCN2 controlling SG assembly and/or translation. It sounds from their discussion as if nothing is really known about GCN2 phophotargets beyond eIF2a and the tRNA synthetase MRS. This latter target could thus presumably be examined to see if it exerts effects on SG assembly also (I understand their arguments as to why this is likely not ternary complex related, but maybe it reflects some other function??).

© 2020. Published by The Company of Biologists under the terms of the Creative Commons Attribution License (https://creativecommons.org/licenses/by/4.0/).

We agree that probing the possible role of MRS in UV-induced SG formation is interesting. However, we believe it is beyond the scope of this work. We plan to examine contribution of MRS to UV-induced SG formation and screen for other potential GCN2 targets in a follow up study.

Alternatively, if other known targets or GCN2 phosphoproteomic datasets don't exist, the authors could also generate their own GCN2-dependent phosphoproteomic dataset, +/- their GCN2 inhibitor (or try a GCN2 substrate pull down in presence of crosslinkers - https://pubmed.ncbi.nlm.nih.gov/30636329/); this would surely be a useful resource to the signaling/GCN2 community. Note, I clearly realize that either of these suggestions is a substantial body of work potentially (especially at this time), thus I don't insist on the authors doing this; merely a suggestion for future work. Also characterizing any hits obtained in their own, or work of others in any detail wouldn't be critical if they did go down this route.

We think that identification of a more complete set of GCN2 targets in addition to eIF2alpha and MRS, which to our knowledge have not been done to date, would be an exciting and useful undertaking. We considered potential approaches for doing this and thank Reviewer 2 for suggesting using a crosslinker. As this type of analysis would be indeed a substantial body of work, it is beyond the scope of this study and we are planning to screen for new GCN2 targets in the future work.

More doable but still informative: Is the Gcn2 kinase in SGs under UV stress? Does its kinase activity affect SG assembly rates, disassembly rates, or both (could do stress timecourse experiments with the inhibitor). Basically, any further foray in the direction of what GCN2 is doing would be an improvement.

We thank Reviewer 2 for this suggestion and agree that more insights into the role of GCN2 activity in UV-induced SG dynamics would improve our manuscript. In the revised version, we provide additional analyses of SG formation dynamics in GCN2-deficient cells by adding another timepoint (6 h, Fig. 4A) and by observing SG formation over time in live cells expressing EGFP-tagged G3BP1 protein with or without treatment with the GCN2 inhibitor (Fig. S2). These analyses revealed that in post-mitotic cells (those that were irradiated during mitosis or within approximately 30 minutes before or after cell division) SG formation occurred within first 2-3 h and did not require GCN2 activity. By contrast, cells that had entered interphase prior to UV exposure formed SGs later (2-6 h post-UV) and their formation was GCN2-dependent. These cells did not form SGs when GCN2 was not expressed or inhibited using A-92 even at 6 h post- UV exposure. In the revised manuscript we also demonstrate that neither total nor phosphorylated GCN2 accumulate in UV-induced SGs (Fig. 4D,E).

3.) Another focus for giving the paper more novel data would be to test the importance of SG assembly using their clever Dox-inducible USP10 system to cell survival under UV stress. Alternatively, they could examine other UV or SG- relevant phenotypes such as proper regulation of UV-responsive mRNAs, signaling events, DNA damage assays etc under UV stress, in the presence or absence of SG assembly.

In the present study, similar to other studies analysing SG formation, we focused on early events. Previous studies analysed the impact of eIF2alpha phosphorylation on UV-induced cell death. We observed that following 20 mJ/cm2 treatment up to 40% of A549 cells round up and detach from the dish at 24 h post-exposure. Aulas et al. (2017) observed only 20% cell death at 24 h post-exposure in HAP1 cells using the same dose. Now that we discovered robust tolls for specifically modulating SG formation post-UV exposure (e.g. USP10(1-40) overexpression, GCN2 inhibition, G3BP1 deletion), we intend to directly assess contribution of SGs to cell cycle arrest and survival in a follow up study with varying dose of UV. These experiments are beyond the scope of the present manuscript that focuses specifically on SG formation. The functional role of SG condensation in stress responses represents one of the main research themes in our laboratory, and it is our goal to analyse contribution of SG formation to cell survival and other phenotypes in future work.

Note, since mRNAs may not be a major part of UV SGs, based on Ivanov's poly(A) staining data and the authors own 4F complex data, the survival test is probably the simpler assay. However, I might

© 2020. Published by The Company of Biologists under the terms of the Creative Commons Attribution License (https://creativecommons.org/licenses/by/4.0/).

suggest the authors check for the presence of de(A) RNA in UV SGs using RNA dyes like SYTO select, just incase de(A) mRNAs are actually what is being regulated (might explain why 4G is absent). The fact that UV SGs do show sensitivity to CHX (Aulas et al, Fig 4A) suggest to me at least that some mRNAs are cycling through UV SGs.

UV-induced SGs are not unique in that they lack easily-detectable poly(A) RNA (for example RocA-induced SGs also do not accumulate poly(A) RNA). We agree with the Reviewer that it is possible that UV-induced condensates accumulate deadenylated mRNAs. However, in our experience SYTO RNA Select does not reliably detect RNA in SGs, including large arsenite- induced SGs. To convincingly demonstrate that deadenylated RNAs are specifically recruited to UV-induced granules, a much more focused study would be required, which is beyond the scope of the present manuscript.

Regarding points 2 and 3 above, my suggestions for minimal revisions/novel additions would be to try the survival and RNA staining experiments in point 3 above (compare the latter to Arsenite SGs?). One of the “GCN2 in SGs / Effects of GCN2 inhibition on SH assembly v disassembly timecourse experiments” (point 2) would be sufficient also. However, going deeper experimentally as I’ve suggested, or other things along these lines would certainly be welcome if the authors are motivated/able to try under current circumstances.

We thank Reviewer 2 for helpful suggestions. We believe that additional experiments presented in the revised version further support our novel findings and improve the manuscript.

Minor points

Certain microscopy and western blots not currently quantitated should be (or in one case, raw data not shown); some discussed in more detail below but to summarize:

Fig 1C (Microscopy); Fig 1D/A (either or; primarily to assess non-P and p-4E-BP levels, though eIF2a, S6K etc also could be quantified); Fig 2B; gels shown, but not quantification; Fig 2C (quantification shown but not actual gels); Fig 2D (Microscopy), Fig 3F (Microscopy), Fig 4D (Microscopy); Fig 5A (Microscopy)
– any/all of these could suffice as supplementary material.

We did not aim at quantifying phosphorylation of eIF2alpha or other phosphoproteins because these blots served as qualitative indicators of phosphorylation status and we did not attempt to corelate signal strength to the magnitudes of any of the measured outcomes. In the revised version, all relevant quantifications of SG formation were included in Figs. 1D, 2D, 4B, 5C, and 5F.

line 48: I would soften/alter statement to reflect idea that some type of non- standard 48S pre-initiation complex may exist in SGs. As the authors likely know, the notion that SGs harbor 48S pre-initiation complexes is not based on any actual knowledge of the mRNP state for any given mRNA, simply the fact that various initiation factor proteins are present in SGs. Additionally, the ref cited (Kedersha et al, 2002, MBC) makes the point that arsenite SGs lack eIF2, hence if 48S complexes of a sort are present, they are already non-canonical by absence of this complex.

We softened the statement on line 48. It now reads: “Consequently, components of the 48S pre-initiation complexes become the major constituents of SGs that form”.

Fig1 A, line 235: I indeed see a 3rd higher molecular weight Phospho band for 4E-BP following UV as authors state; however overall level of phosphorylation appears lower, at least in 1A. This could mean more 4E-BP may now be de-phosphorylated and thus competent for translation repression (meaning TOR would potentially play a role in UV stress translation repression); alternatively 4E-BP levels could simply be reduced? The authors should assess this with a regular 4E-BP Ab western.

We included total protein blots for S6 and 4E-BP, as well as an additional control (arsenite treatment) in the new panel 1A. This clearly indicates that 4E-BP banding pattern changes caused by UV exposure or arsenite treatment are very similar. Furthermore, quantification of UV-induced SGs revealed that mTOR inhibition decreases SG formation post-UV exposure, indicating that the mechanism of SG formation does not rely on mTOR inhibition.

© 2020. Published by The Company of Biologists under the terms of the Creative Commons Attribution License (https://creativecommons.org/licenses/by/4.0/).

Note, in Fig 1D (lanes 1 and 2), it actually looks to me as though total 4EBP levels increase under UV stress (cumulative band intensity for 4EBP in UV v control v actin as normalization). Perhaps the authors could quantitate from this gel (normalized to actin), or simply conditions in 1A with 4E-BP staining. Either way, I think it's important to quantify here.

We repeated these experiments multiple times and have not seen increase in total 4E-BP following UV treatment. Depending on its phosphorylation status, 4E-BP is detected as 4 distinct bands that may appear more or less intense relative to each other. This makes reliable quantitation of signal intensity difficult. However, in the new panel 1A, total 4E-BP banding pattern is similar in Torin-1 treated lanes between different conditions (untreated, UV, and As), which makes it easier to interpret and the total 4E-BP levels do not appear to increase upon UV exposure.

Line 247, Fig 1C: Minor point, but why did authors suspect TOR inhibition might "prevent" UV SG assembly; couldn't it equally have enhanced? Does it? (i.e. are there any quantitative differences in SG number, size etc for SG foci shown in 1C?). More generally, authors may want to clearly state their experiment also assesses whether TOR inhibition per se, often heralded as a master regulator of translation, is capable of inducing SGs; obviously the answer is no.

We thank Reviewer 2 for a helpful suggestion to quantify SG formation in this figure. Quantification of UV-induced SGs revealed that mTOR inhibition decreases SG formation post- UV exposure.

Line 259: Minor point, can ignore but I found wording here confusing. I would just edit to be more explicit and say that 4G no longer binds/pulls down with m7GTP cap, reflecting impairment of 4E-4G interaction. "..dissociation of 4E and 4G.." confused me because obviously 4E doesn't dissociate from the m7GTP cap, which is the basis of the assay, which isn't looking at eIF4G-eIF4E interaction directly.

We have modified the text accordingly. The sentence now reads "As expected, mTOR inhibition by amino acid starvation or Torin-1 caused dephosphorylation of 4E-BP, its association with eIF4E, and inhibited binding of eIF4G to the m7GTP beads, reflecting impairment of eIF4E-eIF4G interaction".

Line 276, Fig 2B: Based on authors own data (and later more accurate statement), I'd soften conclusion on line 276 to "largely" phospho-eIF2a independent.

We have modified the text accordingly.

Line 89/Figure 2D: It would strengthen author's conclusion (e.g. "just as well") if actual quantitation of SG foci was presented rather than just images. This could be cells with foci (as in 3E), average number of cells with foci, intensity, size etc; I leave it up to the authors to choose the most practical, informative measure.

Unlike in U2OS or A549 cells, in MEFs much fewer cells form SGs after UV exposure. We now included quantification of SGs in panel 2D. As Reviewer 2 pointed out, results presented in this figure are not novel and UV-induced SG formation in S51A-eIF2alpha MEFs and HAP1 cells was demonstrated previously (Moutaoufik et al, 2014; Aulas et al, 2017).

Figure 3 / siRNA 1 for GCN2: While very honest, I wonder if it serves the authors to show data where two KDs for GCN2 have different effects (3B, E and F), especially since siRNA 1 already exhibits slow growth and protein synthesis, before the addition of stress etc. I would suggest they just show siRNA KD 2, unless they are able to determine anything mechanistically about why their siRNA1 data is distinct.

We agree that our attempt to be thorough in the description of commercially available siRNA constructs could be perceived as misleading. Accordingly, we removed results for siRNA-1 from the Figure 3 and added new figure (Figure 4) describing experiments using complete GCN2 knock out line that we generated using CRISPR/Cas9 technology. However, we feel that it is important to report the off-target effects of the GCN2 siRNA-1 to the field and retained the original Figure 3 as

© 2020. Published by The Company of Biologists under the terms of the Creative Commons Attribution License (https://creativecommons.org/licenses/by/4.0/).

a supplementary figure (Fig. S1).

I would consider quantifying Figure 4D. Also, does G3BP1 show any particular perinuclear re-localization in UV stress with USP10 peptide expression; sort of looks like this in images shown, but may just be unrepresentative.

We now quantified SG formation in cells expressing our EGFP constructs (new panel 5F). We noticed that in some cells EGFP-USP10(1-40) has somewhat perinuclear localization and consequently recruits G3BP1 to that area. However, this staining pattern does not corelate with SG inhibition by overexpression of this construct.

Figure 5A: It does appear that cells lacking G3BP1 are impaired in UV SG assembly, relative at least to Se and Arsenite treated cells; however, fewer UV SGs form relative to Arsenite stress in the first place (e.g. see Fig 6A).
Thus, it would certainly help if the authors compare to a WT cell control subject to UV stress to make clear to what extent G3BP1-deficient cells are impaired in SG assembly. Some arbitrary threshold could be used to focus on quantifying the cells most depleted in G3BP1 for this purpose. Additionally, because this experimental KO approach likely yields a mixed population of cells that still express G3BP1 or 2, it makes interpreting the G3BP2 KO data more difficult if the authors can't actually visualize G3BP2. They state for example the Se stress results in no SGs forming in G3BP2 KO cells; does this mean the presumed small population of WT cells in this culture are deficient in Se-SG induction due to neighboring cell effects? Or is the G3BP2 KO actually much more efficient? If the authors can't discriminate, I'd either acknowledge these issues, or simply not show the G3BP2 KO microscopy data.

In the revised manuscript we present analysis of G3BP2 KO cell clone (Fig. 5). Despite having decreased levels of G3BP1 as well, these cells are not impaired in UV-induced SG formation. By contrast, G3BP1 KO cells have increased levels of G3BP2 expression yet have impaired SG formation following UV exposure.
The original panel 5A has been replaced with these new analyses and quantitation in the revised manuscript.

Second decision letter

MS ID#: JOCES/2020/248310

MS TITLE: UV damage induces G3BP1-dependent stress granule formation that is not driven by translation arrest via mTOR inhibition

AUTHORS: Shan Ying and Denys Khaperskyy
ARTICLE TYPE: Research Article

We have now reached a decision on the above manuscript.

To see the reviewers' reports and a copy of this decision letter, please go to: https://submit-jcs.biologists.org and click on the 'Manuscripts with Decisions' queue in the Author Area. (Corresponding author only has access to reviews.)

As you will see, the reviewers recognize that many initial concerns have been addressed in your revised manuscript. However, reviewer #2 still raised criticisms that will require further amendments to your manuscript. Note that no further experiments are requested at this stage. I hope that you will be able to discuss in a revised version the issues raised by reviewer #2, because I would like to be able to accept your paper.

We are aware that you may currently be unable to access the lab to undertake experimental revisions. If it would be helpful, we encourage you to contact us to discuss your revision in greater

© 2020. Published by The Company of Biologists under the terms of the Creative Commons Attribution License (https://creativecommons.org/licenses/by/4.0/).

detail. Please send us a point-by-point response indicating where you are able to address concerns raised (either experimentally or by changes to the text) and where you will not be able to do so within the normal timeframe of a revision. We will then provide further guidance. Please also note that we are happy to extend revision timeframes as necessary.

Please ensure that you clearly highlight all changes made in the revised manuscript. Please avoid using 'Tracked changes' in Word files as these are lost in PDF conversion.

I should be grateful if you would also provide a point-by-point response detailing how you have dealt with the points raised by the reviewers in the 'Response to Reviewers' box. Please attend to all of the reviewers' comments. If you do not agree with any of their criticisms or suggestions please explain clearly why this is so.

Reviewer 1

Advance summary and potential significance to field

This paper describes how UV-induced granules are dependent on G3BP1, but not G3BP2, via GCN2 pathway. It confirms and extends work in the field.

Comments for the author

The authors have incorporated the changes suggested by both reviewers in the previous round and is suitable for publication, in my opinion. Indeed, the manuscript is greatly improved with the addition of the new data requested.
Addition of GCN2-ko cells is appreciated inlight of the disparate results with siRNA approach. It confirms some pre-existing work on UV-induced stress granules and extends our knowledge about the underlying mechanism.

Reviewer 2

Advance summary and potential significance to field

The authors have done a reasonable job in their revision, though there are some gaps. I think the paper, at minimum, still requires some minor writing/quantitation revisions that should be straightforward. There are a couple of opportunities for discussing data more that I leave at the authors discretion. My concerns on novelty and mechanistic understanding mostly remain. As reviewer two noted previously, the two main novel aspects of the paper are that UV-induced SGs require GCN2 and G3BP1 is involved. However we knew GCN2 was required for translation repression following UV stress from Aulas et al, JCS in 2017, thus it was not a huge stretch to presume SG assembly would be affected also (given SGs rarely, if ever form without translation repression) – importantly we still don't know how GCN2 drives UV SG assembly. G3BP1 is also the most commonly implicated SG assembly factor.
However the work is otherwise high quality and so I am modestly in favor of publication if the writing and quantitation issues are addressed.

Comments for the author

Remaining concerns/unaddressed issues:
I still have concerns about novelty and how much new we have learned for the reasons stated in my previous review (Yes negative data is useful, but less so than actual mechanistic understanding).
On the plus side, the authors have done a good job on addressing my requests about SG assembly dynamics under UV stress, and in the process discovered a new lead relating cell cycle positioning to the effect of UV, and GCN2, on SG induction. Confirming GCN2 is not in SGs is also helpful.
Adding PKR control data (suggested by the other reviewer) also strengthens their conclusion a little (but doesn't change it).
On the negative side, while some of my suggestions were indeed fairly broad (e.g. identifying the meaningful GCN2 Phospho target, investigate MRS), others that seemed "easy" to me for the

© 2020. Published by The Company of Biologists under the terms of the Creative Commons Attribution License (https://creativecommons.org/licenses/by/4.0/).

authors to attempt included using the USP10 SG impairing system to see whether the inability to form SGs has any phenotypic effects following UV stress (e.g. altered survival, which evidently in their rebuttal response they've already assessed for eIF2a phospho effects). In other words, something to say that formation of these UV SGs actually matters. They also at several points in the manuscript refer to mRNPs in UV SGs when in fact there is currently little or no evidence that (m)RNA is even in these SGs. The authors state they have had problems getting SYTO to work but provide no evidence of their attempts – in our hands it has worked fine – but if so, then try acridine orange, or some other nucleic acid dye (DAPI staining will remove doubt of what is DNA v RNA). Because I asked the authors to attempt these experiments as part of my minimal revision, and they haven't (for scope/technical reasons), on the one hand I feel they could have gone further. However, because the paper is otherwise sound, and because COVID-19 is limiting experiments, I will not insist on these experiments if the editor is satisfied.

Also, while I get that western blots are not completely quantitative as presented in most papers, and while the authors do not correlate band intensity strength to magnitude of outcomes, they do in many places make "binary" conclusions – there is no difference, or there is. Where those conclusions are made, in my view, they would be better substantiated by quantitation. Because we are looking at a single representative image, it's also hard to know, without such quantitation, how variable (if at all) the banding patterns are. My experiences are likely colored by the fact that every paper we've submitted with western blotting in the past 5 years has required all bands be quantified that we discuss.

New data issues:
1. Figure 1D: While the initial conclusions drawn are fair, the observations that inhibiting TOR, or depriving of amino acids, actually impairs UV induction of SGs are odd. The former result suggests TOR activity may in fact stimulate UV-induced SG formation – presumably this is not likely to be related to the degree of translation repression given 1B (i.e. further evidence of disconnect between translation repression and SG assembly). If the authors have any speculation on why TOR activity might aid SG induction following UV stress, I would add it in the discussion (around line 530).
The latter result is also odd because aa deprivation will activate GCN2 signaling (which the authors model suggests is required for UV SG induction), thus why fewer SGs forms is presumably related to some other effect, such as mTOR inhibition? Again, can maybe briefly discuss, though if the authors have no idea I will let this slide.

2. Fig 4A/Line 400: By "Parental", do the authors mean to say cell that have recently undergone cell division? If so, the latter term would be clearer.
Otherwise, I don't follow their description.

Additional minor suggestions: (apologies for not mentioning some of these comments on the first round, but they should only necessitate writing changes/minor quantitation).
1. The title and abstract are disconnected (no mention of mTOR data in the abstract at all). I would have the title focused on UV SGs being driven by GCN2 in an eIF2a-independent, G3BP1 dependent manner.

2. I would add details on the dose of UV used in different experiments in the figure legends where not explicitly mentioned in the figure or results text itself (although methods say 10 or 20 mJ/cm2 on line 125, it would be helpful to know what was used in each experiment as Fig 1B indicates there is a difference in magnitude of effect on translation).

3. Fig1 B/Lines 278-281: I previously asked that quantitation be added for this figure (as was done for other ribopruomycylation assays in the paper) – I'd still like this done. Additionally, the authors statement could be clearer and more explicit here – it sounds like they are trying to say UV inhibition does not result in the same degree of translation inhibition as with Torin-1 treatment. By eye (again imperfect without quantitation), lanes 2 and 3 look very similar to me (Torin1 and 10 mJ/cm2 UV respectively), whereas 20 mj/cm2 UV (lane 5) looks to more strongly inhibit translation. I'd make it clearer that UV stress can inhibit translation more than mTOR inhibition at higher doses, if that's in fact the point the authors really mean to make.

© 2020. Published by The Company of Biologists under the terms of the Creative Commons Attribution License (https://creativecommons.org/licenses/by/4.0/).

4. Fig 2B: Ditto the above point (previously requested quantitation, not done). Although the effect looks clear, since quantiation has been done for other ribopuromycinylation assays in the paper, it just seems inconsistent to leave out in certain figures (also quants give more info with error significance tests etc).

5. Fig S1E (old Fig 3E) – Still not quantitated.

6. The discussion is still rather long – personally I would cut the ATF4 stuff (around lines 560), especially because at this point we don't have any firm evidence that mRNAs are actually in UV stress granules, thus it's unclear if UV stress is doing anything to mRNA fate, in a SG-dependent manner. I'd also cut much in the UV SGs differ from canonical SGs section (line 615-661) since it's mostly just saying that the authors data is the same as the previous Ivanov study and/or doesn't clearly highlight what differences they say, or simply re-describes the methods/results. Ultimately though, if the authors wish to keep the discussion as is, I won't object.

Second revision

Author response to reviewers' comments

Reviewer 1 Advance Summary and Potential Significance to Field:
This paper describes how UV-induced granules are dependent on G3BP1, but not G3BP2, via GCN2 pathway. It confirms and extends work in the field.

Reviewer 1 Comments for the Author:
The authors have incorporated the changes suggested by both reviewers in the previous round and is suitable for publication, in my opinion. Indeed, the manuscript is greatly improved with the addition of the new data requested. Addition of GCN2-ko cells is appreciated inlight of the disparate results with siRNA approach. It confirms some pre-existing work on UV-induced stress granules and extends our knowledge about the underlying mechanism.

We thank reviewer 1 for their comments and appreciate their help in making our paper better. We agree that after the first round of revisions suggested by both reviewers 1 and 2 and addition of new experimental results our manuscript is greatly improved.

Reviewer 2 Advance Summary and Potential Significance to Field:
The authors have done a reasonable job in their revision, though there are some gaps. I think the paper, at minimum, still requires some minor writing/quantitation revisions that should be straightforward. There are a couple of opportunities for discussing data more that I leave at the authors discretion. My concerns on novelty and mechanistic understanding mostly remain. As reviewer two noted previously, the two main novel aspects of the paper are that UV-induced SGs require GCN2 and G3BP1 is involved. However, we knew GCN2 was required for translation repression following UV stress from Aulas et al, JCS in 2017, thus it was not a huge stretch to presume SG assembly would be affected also (given SGs rarely, if ever form without translation repression) – importantly we still don't know how GCN2 drives UV SG assembly. G3BP1 is also the most commonly implicated SG assembly factor.
However the work is otherwise high quality and so I am modestly in favor of publication if the writing and quantitation issues are addressed.

We thank reviewer 2 for their comments and acknowledge that the mechanism thereby GCN2 drives SG formation in interphase cells remains to be characterized and other gaps in knowledge that still exist need to be filled. We plan to tackle these questions in our future studies.

Reviewer 2 Comments for the Author:

Remaining concerns/unaddressed issues:
I still have concerns about novelty and how much new we have learned for the reasons stated in

© 2020. Published by The Company of Biologists under the terms of the Creative Commons Attribution License (https://creativecommons.org/licenses/by/4.0/).

my previous review (Yes negative data is useful, but less so than actual mechanistic understanding). On the plus side, the authors have done a good job on addressing my requests about SG assembly dynamics under UV stress, and in the process discovered a new lead relating cell cycle positioning to the effect of UV, and GCN2, on SG induction. Confirming GCN2 is not in SGs is also helpful. Adding PKR control data (suggested by the other reviewer) also strengthens their conclusion a little (but doesn't change it). On the negative side, while some of my suggestions were indeed fairly broad (e.g. identifying the meaningful GCN2 Phospho target, investigate MRS), others that seemed "easy" to me for the authors to attempt included using the USP10 SG impairing system to see whether the inability to form SGs has any phenotypic effects following UV stress (e.g. altered survival, which evidently in their rebuttal response they've already assessed for eIF2a phospho effects). In other words, something to say that formation of these UV SGs actually matters.

We thank Reviewer 2 for their critical input. We agree that in this study we did not identify the molecular mechanism involved in GCN2-mediated SG nucleation by means of identifying target(s) of GCN2 that contribute to the process. We also did not test effects of SGs on cell cycle arrest or survival post-UV damage due to limited access to flow cytometry core facilities during implementation of pandemic protocols. However, we respectfully disagree that our study lacks novelty or presents only negative data. In our previous response we stated that the focus of our work was on the contribution of mTOR signaling, GCN2 activation, and the role of SG-nucleating proteins in UV-induced SG formation, which to our knowledge had not been systematically analysed previously. In our study, we show for the first time that

1) mTOR pathway remains active after UV exposure and that inhibition of translation and SG formation following UV stress do not require mTOR inhibition;

2) we quantify contribution of GCN2-mediated eIF2alpha phosphorylation to UV-induced translation arrest (similar quantitative analysis has not been performed previously);

3) GCN2 catalytic activity is required for UV-induced SG formation;

4) G3BP1, but not G3BP2, is required for UV-induced SG formation;

5) although eIF4G and eIF3B are not recruited to UV-induced SGs, their association within preinitiation complexes is unaffected in most mRNPs in the cell.

Even though our manuscript features two "confirmatory" findings (UV-induced SG formation is phospho-eIF2alpha independent and eIF4G/eIF3B are not recruited to UV-induced SGs), these do not represent the main conclusions of our study. Therefore, we believe our study is of high quality and rigour and has sufficient novelty to warrant publication in JCS, even if it does not provide complete mechanistic understanding of the phenomena that were not known prior to our study.

They also at several points in the manuscript refer to mRNPs in UV SGs when in fact there is currently little or no evidence that (m)RNA is even in these SGs. The authors state they have had problems getting SYTO to work but provide no evidence of their attempts – in our hands it has worked fine – but if so, then try acridine orange, or some other nucleic acid dye (DAPI staining will remove doubt of what is DNA v RNA). Because I asked the authors to attempt these experiments as part of my minimal revision, and they haven't (for scope/technical reasons), on the one hand I feel they could have gone further.
However, because the paper is otherwise sound, and because COVID-19 is limiting experiments, I will not insist on these experiments if the editor is satisfied.

We agree that demonstrating whether RNA is present in UV-induced SGs would have value. However, without knowing the nature of the RNA signal generated by nucleic acid dyes one cannot speculate that those are indeed deadenylated mRNAs or any other type of RNA (rRNA, long non-coding RNA, endogenous retroviral RNA, to name a few). Therefore, in this revision we could not dedicate additional time and resources to acquire, test, and troubleshoot multiple nucleic acid dyes to simply demonstrate the presence or absence of nucleic acid in UV-induced SGs (speculatively, some RNA is likely to be present in SGs as RNA has been shown by multiple studies to be an important driver of phase separation under physiological conditions). In future, it would

© 2020. Published by The Company of Biologists under the terms of the Creative Commons Attribution License (https://creativecommons.org/licenses/by/4.0/).

be desirable to conduct a more focused study using an array of molecular techniques to compare RNA composition in various types of SGs for which poly(A)/PABP recruitment was shown to be weak or absent.

Also, while I get that western blots are not completely quantitative as presented in most papers, and while the authors do not correlate band intensity strength to magnitude of outcomes, they do in many places make “binary” conclusions – there is no difference, or there is. Where those conclusions are made, in my view, they would be better substantiated by quantitation. Because we are looking at a single representative image, it’s also hard to know, without such quantitation, how variable (if at all) the banding patterns are. My experiences are likely colored by the fact that every paper we’ve submitted with western blotting in the past 5 years has required all bands be quantified that we discuss.

As we stated in our previous response, we did not aim at quantifying phosphorylation of eIF2alpha or other phosphoproteins because these blots served as qualitative indicators of phosphorylation status and we did not attempt to corelate signal strength to the magnitudes of any of the measured outcomes. We believe that addition of western blot quantifications at this point would add more clutter to the figures without providing substantial insight. In our experience, reviewers seldom ask for western blot band quantification, unless subtle changes in the levels of proteins are one of the major points of the study.

New data issues:

1. Figure 1D: While the initial conclusions drawn are fair, the observations that inhibiting TOR, or depriving of amino acids, actually impairs UV induction of SGs are odd. The former result suggests TOR activity may in fact stimulate UV-induced SG formation – presumably this is not likely to be related to the degree of translation repression given 1B (i.e. further evidence of disconnect between translation repression and SG assembly). If the authors have any speculation on why TOR activity might aid SG induction following UV stress, I would add it in the discussion (around line 530). The latter result is also odd because aa deprivation will activate GCN2 signaling (which the authors model suggests is required for UV SG induction), thus why fewer SGs forms is presumably related to some other effect, such as mTOR inhibition? Again, can maybe briefly discuss, though if the authors have no idea I will let this slide.

We now add additional discussion of possible contribution of mTOR to UV-induced SG formation: “One possible explanation for decreased SG formation after mTOR inhibition is induction of autophagy – the catabolic process proposed to accelerate SG disassembly (Monahan et al., 2016; Protter and Parker, 2016). Alternatively, SG formation following UV damage may require initial influx of intact 48S translation preinitiation complexes that become modified as part of the mechanism of SG condensation.”

2. Fig 4A/Line 400: By “Parental”, do the authors mean to say cell that have recently undergone cell division? If so, the latter term would be clearer. Otherwise, I don’t follow their description.

Words “parental” in the paragraph (lines 391, 396) refer to U2OS cells that were used to generate GCN2 knockout clones. To clarify we included “GCN2 knock out” in the phrase on line 395: “Majority of GCN2 knock out cells remained SG-free even at later 6 h timepoint, while more than half of all the parental U2OS cells formed SGs (Fig. 4A).”

Additional minor suggestions: (apologies for not mentioning some of these comments on the first round, but they should only necessitate writing changes/minor quantitation).

1. The title and abstract are disconnected (no mention of mTOR data in the abstract at all). I would have the title focused on UV SGs being driven by GCN2 in an eIF2a-independent, G3BP1 dependent manner.

We would prefer to avoid modifications to the title at this point. The latest revision now has reference to mTOR in the abstract.

2. I would add details on the dose of UV used in different experiments in the figure legends where not explicitly mentioned in the figure or results text itself (although methods say 10 or 20 mJ/cm2

© 2020. Published by The Company of Biologists under the terms of the Creative Commons Attribution License (https://creativecommons.org/licenses/by/4.0/).

on line 125, it would be helpful to know what was used in each experiment as Fig 1B indicates there is a difference in magnitude of effect on translation).

We now added UV dose to all figure legends.

3. Fig1 B/Lines 278-281: I previously asked that quantitation be added for this figure (as was done for other ribopruomycylation assays in the paper) – I'd still like this done. Additionally, the authors statement could be clearer and more explicit here – it sounds like they are trying to say UV inhibition does not result in the same degree of translation inhibition as with Torin-1 treatment. By eye (again imperfect without quantitation), lanes 2 and 3 look very similar to me (Torin1 and 10 mJ/cm2 UV respectively), whereas 20 mj/cm2 UV (lane 5) looks to more strongly inhibit translation. I'd make it clearer that UV stress can inhibit translation more than mTOR inhibition at higher doses, if that's in fact the point the authors really mean to make.

We now include quantification in new panel 1C. We also slightly modified description in the results section to make our conclusions from these data clearer.

4. Fig 2B: Ditto the above point (previously requested quantitation, not done). Although the effect looks clear, since quantiation has been done for other ribopuromycinylation assays in the paper, it just seems inconsistent to leave out in certain figures (also quants give more info with error, significance tests etc).

We now include quantification of panel 2B in the new figure S1.

5. Fig S1E (old Fig 3E) – Still not quantitated.

We now include SG quantification in panel S1E.

6. The discussion is still rather long – personally I would cut the ATF4 stuff (around lines 560), especially because at this point we don't have any firm evidence that mRNAs are actually in UV stress granules, thus it's unclear if UV stress is doing anything to mRNA fate, in a SG-dependent manner. I'd also cut much in the UV SGs differ from canonical SGs section (line 615-661) since it's mostly just saying that the authors data is the same as the previous Ivanov study and/or doesn't clearly highlight what differences they say, or simply re-describes the methods/results. Ultimately though, if the authors wish to keep the discussion as is, I won't object.

We agree that there is no firm evidence that mRNAs are actually in UV stress granules. However, our Cap-pulldown and co-immunoprecipitation analyses clearly show that most mRNPs remain intact and therefore UV stress in general and eIF2a phosphorylation in particular could have effects on those mRNPs. We also would like to keep "the UV SGs differ from canonical SGs section (line 615-661)" because in our view discussion refers to "discussion of our results in the context of existing literature", including the Ivanov study.

Third decision letter

MS ID#: JOCES/2020/248310

MS TITLE: UV damage induces G3BP1-dependent stress granule formation that is not driven by translation arrest via mTOR inhibition

AUTHORS: Shan Ying and Denys Khaperskyy
ARTICLE TYPE: Research Article

I am happy to tell you that your manuscript has been accepted for publication in Journal of Cell Science, pending standard ethics checks.

© 2020. Published by The Company of Biologists under the terms of the Creative Commons Attribution License (https://creativecommons.org/licenses/by/4.0/).
